# Exploring Parameter and Hyper-Parameter Spaces of Neuroscience Models on High Performance Computers With Learning to Learn

**DOI:** 10.3389/fncom.2022.885207

**Published:** 2022-05-27

**Authors:** Alper Yegenoglu, Anand Subramoney, Thorsten Hater, Cristian Jimenez-Romero, Wouter Klijn, Aarón Pérez Martín, Michiel van der Vlag, Michael Herty, Abigail Morrison, Sandra Diaz-Pier

**Affiliations:** ^1^Simulation and Data Lab Neuroscience, Jülich Supercomputing Centre (JSC), Institute for Advanced Simulation, JARA, Forschungszentrum Jülich GmbH, Jülich, Germany; ^2^Department of Mathematics, Institute of Geometry and Applied Mathematics, RWTH Aachen University, Aachen, Germany; ^3^Institute of Neural Computation, Ruhr University Bochum, Bochum, Germany; ^4^Institute of Neuroscience and Medicine (INM-6), Institute for Advanced Simulation (IAS-6), JARA BRAIN Institute I, Jülich Research Centre, Jülich, Germany; ^5^Computer Science 3-Software Engineering, RWTH Aachen University, Aachen, Germany

**Keywords:** simulation, meta learning, hyper-parameter optimization, high performance computing, connectivity generation, parameter exploration

## Abstract

Neuroscience models commonly have a high number of degrees of freedom and only specific regions within the parameter space are able to produce dynamics of interest. This makes the development of tools and strategies to efficiently find these regions of high importance to advance brain research. Exploring the high dimensional parameter space using numerical simulations has been a frequently used technique in the last years in many areas of computational neuroscience. Today, high performance computing (HPC) can provide a powerful infrastructure to speed up explorations and increase our general understanding of the behavior of the model in reasonable times. Learning to learn (L2L) is a well-known concept in machine learning (ML) and a specific method for acquiring constraints to improve learning performance. This concept can be decomposed into a two loop optimization process where the target of optimization can consist of any program such as an artificial neural network, a spiking network, a single cell model, or a whole brain simulation. In this work, we present L2L as an easy to use and flexible framework to perform parameter and hyper-parameter space exploration of neuroscience models on HPC infrastructure. Learning to learn is an implementation of the L2L concept written in Python. This open-source software allows several instances of an optimization target to be executed with different parameters in an embarrassingly parallel fashion on HPC. L2L provides a set of built-in optimizer algorithms, which make adaptive and efficient exploration of parameter spaces possible. Different from other optimization toolboxes, L2L provides maximum flexibility for the way the optimization target can be executed. In this paper, we show a variety of examples of neuroscience models being optimized within the L2L framework to execute different types of tasks. The tasks used to illustrate the concept go from reproducing empirical data to learning how to solve a problem in a dynamic environment. We particularly focus on simulations with models ranging from the single cell to the whole brain and using a variety of simulation engines like NEST, Arbor, TVB, OpenAIGym, and NetLogo.

## 1. Introduction

An essential common tool to most efforts around brain research is the use of algorithms for analysis and simulation. Specialists have developed a large variety of tools that typically rely on many parameters in order to produce the desired results. Finding an appropriate configuration of parameters is a highly non-trivial task that usually requires both experience and the patience to comprehensively explore the complex relationships between inputs and outputs. This problem is common to all input and output formats, as they differ in their type such as images, continuous or discrete signals, experimental data, spiking activity, functional connectivity, etc. In this article, we focus on parameter specification for simulation.

In order to address this problem, we present a flexible tool for parameter optimization: L2L. Initially inspired by the learning to learn (L2L) concept in the machine learning (ML) community, the L2Lframework is an open-source Python tool^1^ that can be used to optimize different workloads. The flexibility of the framework allows the user to set the target of optimization to be a model which can be executed either from Python or the command line. The optimization target can also be adaptive and capable of learning, providing a natural way to carry out hyper-parameter optimization. The L2L framework can be used in local computers as well as on clusters and high performance computing (HPC) infrastructure.

This manuscript is structured as follows. First, we provide a quick overview on the state of the art for optimization methods and highlight the main differences between those tools and the L2L framework. In Section 2, we provide an overview of the framework's architecture, its implementation, and the way it can be used and extended. We then demonstrate its effectiveness on a variety of use cases focused on neuroscience simulation at different scales (Section 3).

### 1.1. State of the Art

In the field of ML, the concept of L2L (c.f. Section 2.1) has been well studied. The L2L concept can be decomposed into two components: (a) an inner loop where a program to be optimized, here named the optimizee, executes specific tasks and returns a measure of how well it performs, called the fitness, and (b) an outer loop where an optimizer searches for generalized optimizee parameters (hyper-parameters) that improve the optimizee's performance over distinct tasks measured by the fitness function. The fitness function is different for each model and tightly linked to the expected transitions in its dynamics. The optimizee can consist of any program such as an artificial neural network, a spiking network, a single cell model, or a whole brain simulation using rate models. In a recent work, Andrychowicz et al. (2016) proposed using long short term memory network (LSTM) with access to the top-level gradients to produce the weight updates for the task LSTM. The main idea is to replace the gradient descent optimizer of the optimizee with an LSTM as an optimizer. In this case, the weights of the inner loop network are treated as the hyper-parameters and trained/learned in the outer loop, while being kept fixed in the inner loop. Based on the work of Andrychowicz et al. (2016) and Ravi and Larochelle (2017) modified the optimization scheme so that the test error can be incorporated into the optimization step. Thus, the optimization can be executed in fewer steps which leads to fewer unrollings of the LSTMs and a reduction of the computational burden. By representing the learning updates of the classifier within the hidden state of the outer-loop optimizer network, the authors acquire a good initialization for the parameters of the inner-loop learner and for further update steps.

For feed-forward networks, Model Agnostic Meta-Learning (MAML) was introduced by Finn et al. (2017). MAML can learn initial parameters for a base-model solving inner-loop-level task. After a few steps of optimization with gradient descent, the base-model can generalize well on the validation set, which is the related data seen for the first time from the same class as the training set. The method can be applied to a vast set of learning problems since the learning itself is agnostic to the inner-loop model. Finn and Levine (2017) showed that learning the initialization combined with gradient updates was as powerful as L2L using a recurrent network. Several extensions have been proposed to enhance the performance of the learning and computation time (Finn et al., 2018, 2019). For example, Li et al. (2017) introduce META-SGD, a stochastic gradient optimization method that not only learns the parameter initialization but also the gradient update of the base-model optimization. However, Antoniou et al. (2018) list several issues found with MAML, such as training instabilities, due to repeated application of backpropagation through the same network multiple times which leads to gradient issues. This leads to a performance drop in learning and computational overhead. A gradient-free version of MAML was proposed by Song et al. (2019) using evolution strategies to replace the second-order backpropagation used in MAML. A framework that is model agnostic but does not depend on calculating gradients or backpropagating through networks and is not limited to a single optimization algorithm would be highly desirable, especially to address the needs of highly interdisciplinary fields such as neuroscience.

Cao et al. (2019) utilize particle swarm optimization (Kennedy and Eberhart, 1995) to train a meta-optimizer that learns both point-based and population-based optimization algorithms in a continuous manner. The authors apply a set of LSTMs to train and learn the update formula for a population of samples. Their learning is based on two attention mechanisms, the feature-level (“intra-particle”) and sample-level (“inter-particle”) attentions. The intra-particle module reweights every feature based on the hidden state of the corresponding *i*-th LSTM, whereas the inter-particle attention module learns in the update step of the actual particle information from the previous already updated particles.

Similarly, Jaderberg et al. (2017) use a parallel population-based approach and random search to optimize the hyper-parameters of neural networks. They randomly sample the initialization of the network parameters and hyper-parameters and every training run is evaluated asynchronously. If a network is underperforming, it is replaced by a more successful network. Furthermore, by perturbing the hyper-parameters of the replacing network the search space is expanded. Neural architecture search (Zoph and Le, 2016) and related methods have been shown to be very useful in choosing network architectures for various tasks. A random search was shown to be surprisingly effective for hyper-parameter searches for a wide variety of tasks (Bergstra and Bengio, 2012). Many of the automated hyper-parameter searches also fall under the category of Automated Machine Learning or AutoML (Hutter et al., 2019; He et al., 2021).

In the area of computational neuroscience, BluePyOpt (Van Geit et al., 2016) has represented a robust solution to address optimization problems. Even if it was originally meant to support the optimization of single cell dynamics, BluePyOpt is also able to optimize models at other scales. It makes use of DEAP (Fortin et al., 2012) for the optimization algorithms and of SCOOP (Hold-Geoffroy et al., 2014) to provide parallelization. The target of optimization in BluePyOpt is also quite flexible, it can be any simulator that can be called from Python. This framework can also be used in different infrastructures, from laptops to clusters. However, the framework only allows the execution of optimization targets written in Python.

Deep Learning compatible spiking network libraries, such as NengoDL (Rasmussen, 2018) or Norse (Pehle and Pedersen, 2021), are getting more popular. They are based on modern tensor libraries and can be executed on GPUs which can speed up the simulations. Although these libraries do not focus on meta-learning they are interesting for solving ML tasks using spiking neural networks (SNN). They can be used to quickly learn the tasks while the hyper-parameters of the SNNs can be optimized in an outer loop.

The L2L framework offers a flexible way to optimize and explore hyper-parameter spaces. Due to its interface, the optimization targets are not restricted to executables with a Python interface offering the possibility to optimize models written in different programming languages. In our work, we focus on neuroscientific use cases. The framework, however, is available for a variety of simulations in different scientific domains. Furthermore, the framework is agnostic to the inner loop models and thus allows for different types of optimization techniques in the outer loop. Most of the optimizers adapt population-based computational algorithms, which enable parallel executions of optimizees (see Section 3). This helps to optimize for a vast range of parameter ranges. The error or rather fitness of the inner loop on the absolved tasks is included in the optimization step to update the parameters. Optimizers such as the genetic algorithm or ensemble Kalman filter (EnKF) use the fitness in order to rank the individuals and replace underperfoming individuals with more successful ones (e.g., see Section 3.1).

## 2. Methods

### 2.1. Concept of L2L

**Figure 1:**
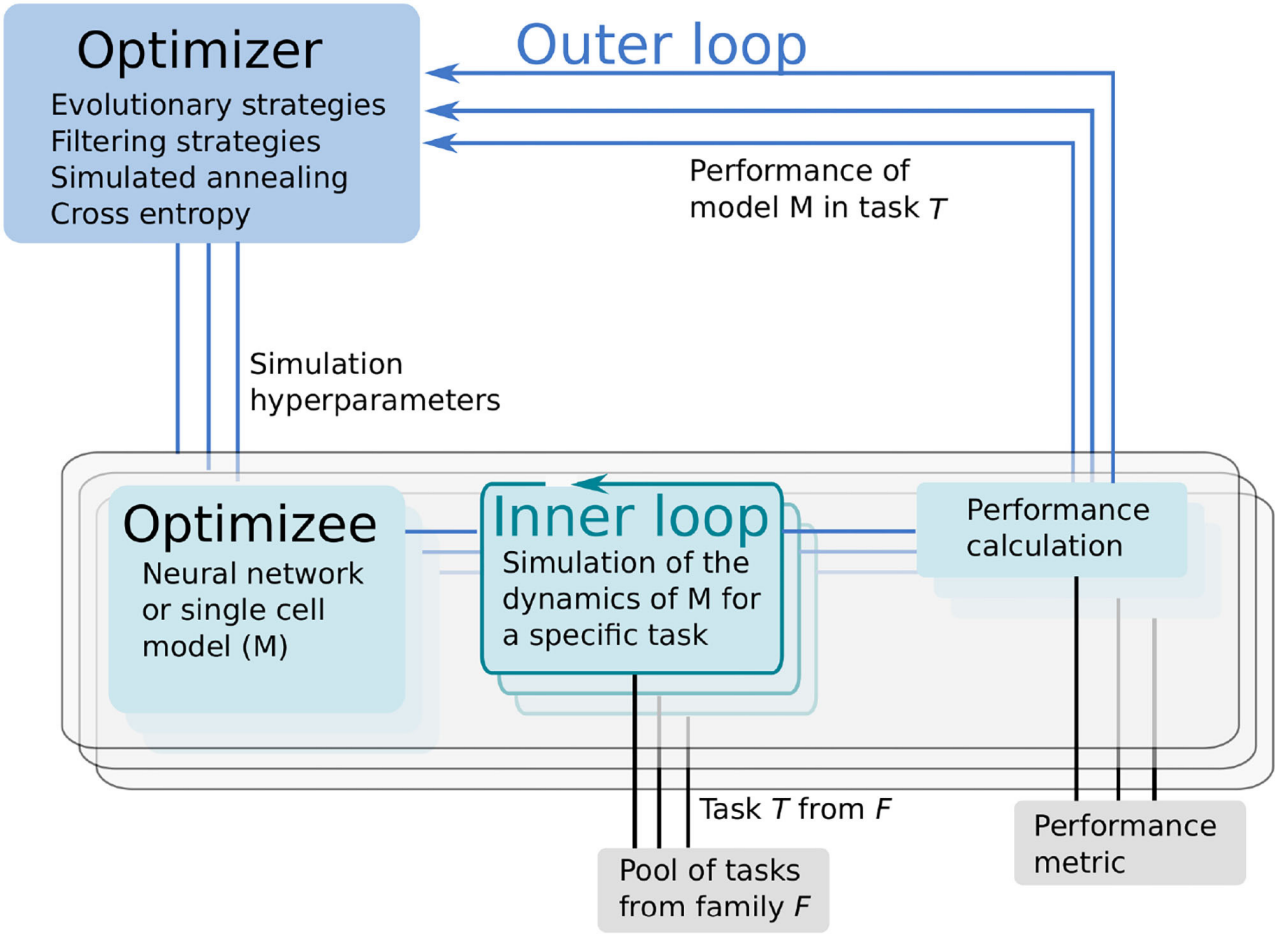
Learning to learn (L2L) consists of two loops. In the inner loop, the optimizee, an algorithm with learning capabilities is trained on a family of tasks. A fitness function evaluates the performance of the algorithm. The (hyper-) parameters and the fitness value of the algorithm are sent to the optimizer in the outer loop. Several optimization methods are available to optimize the parameters, which are fed back to the optimizee and the algorithm.

Learning to learn or meta-learning is a technique to induce learning from experience (Thrun and Pratt, 2012). The L2L process consists of two loops, the inner and outer loop (Figure 1). In the inner loop, an algorithm with learning capabilities (e.g., an artificial or SNN, a single cell model or a whole brain simulation using rate models) is executed on a specific task *T* from a family F of tasks.

Tasks can range from classification (e.g., MNIST; LeCun et al., 2010, see Section 3.1), to identifying parameter regimes that result in specific network dynamics (Sections 3.2, 3.4) or training agents to autonomously solve optimization problems (Sections 3.3, 3.5).

The performance of the algorithm over tasks is evaluated with a specifically designed fitness function, which produces a fitness value *f* or a fitness vector **f**. The function is, in general, different for every model but closely connected to the task itself. Parameters and hyper-parameters, together with the fitness value of the optimizee are sent to the outer loop. Different optimization techniques, such as evolutionary algorithms, filtering methods or gradient descent, can be utilized to optimize the hyper-parameters in order to improve the performance of the optimizee. Afterward, the hyper-parameters are fed back into the algorithm and a new iteration (i.e., a new generation) is invoked. It is important to note that from a technical point of view, the optimizee acts as an orchestrator of the inner loop. Each optimizee executes a simulation. Borrowing the terminology from evolutionary algorithms, the parameter set which is optimized is called an individual. The optimizee accepts (hyper-)parameters from the outer loop and starts the inner loop process to execute the simulation. Last, it calculates the fitness and transmits everything to the optimizer.

### 2.2. Parallel Executions in the L2L Framework

In L2L, the optimizers apply population based methods which enable simulations to be run in an embarrassingly parallel fashion. Each individual is initialized independently. They can be easily distributed on several computing nodes and thus can exploit HPC systems. The L2L framework supports the message passing interface (MPI) over several nodes and multi-threading per node. The number of nodes and cores can be set in the beginning of the run and the L2L framework will automatically take care of the distribution and collection of results. Section 2.3 explains in detail how to set up a simulation run in L2L.

### 2.3. Workflow Description

In L2L, the user has to work on two main files. The first file is the **run script**, which invokes the whole L2L two loop run. The second file is the **optimizee**, which operates the simulation in the inner loop.

In the run script, the user configures hardware-related settings, e.g., if the run is executed on a local computer or on an HPC. Furthermore, the optimizee and optimizer and their parameter options have to be set. An example code template to start the whole L2L run is shown in Listing 1. Lines 1-3 import the necessary modules, i.e., the experiment, optimizee, and the optimizer. Of course, in the real run, the names of the modules and classes have to be adapted to their respective class names, for simplicity, we call them here optimizee and optimizer. The **experiment** class manages the run. In line 5, the results path is set in the constructor of the class. The experiment method prepare_experiment in line 7 prepares the run. It accepts the name of the run, whether logging should be enabled, and the Juelich Benchmarking Environment (JUBE; Speck et al., 2021) parameters. In L2L, JUBE's functionality was stripped down to submit and manage parallel jobs on HPCs and interact with the job management system SLURM (Yoo et al., 2003). The execution directives for the HPC jobs can be seen in line 6. Here, exec is the indicator command to invoke a run on a supercomputer, followed by a srun directive for SLURM. In the example, one task (-n 1) should be run on 8 cores (-c 8). Optimizees and optimizers run as Python executables, which is why the python command is needed here. If a local run is desired, just the Python command is sufficient, i.e., “exec”:“python.” Internally, JUBE creates a job script and passes it to SLURM, which then executes the parallel optimizees and the optimizer. JUBE accepts many more commands for SLURM, but elaborating on all options would go beyond the scope of this work; see the SLURM documentation^2^ for a list of executives. The run script can be executed either as a batch script or as an interactive job on an HPC.

**Listing 1:**
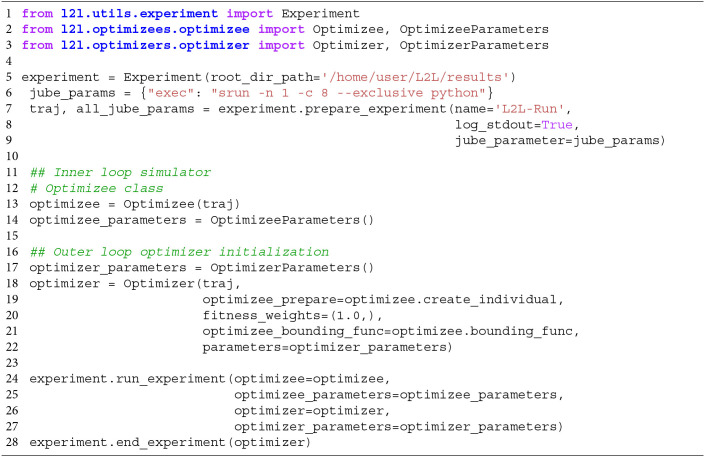
Template script to start a L2L run. The optimizee, optimizer are defined. The experiment class is managing the run.

The **optimizee** is defined in line 13 and requires only the trajectory traj. The trajectory, modeled after PyPet's trajectory^3^, is a class that holds the history of the parameter space exploration and the results from each execution and the parameters to be explored. OptimizeeParameters is a Python namedtuple object, which accepts the parameters of the optimizee. For the optimizee, the namedtuple appears as a parameter object and can be accessed as a class variable, i.e., as parameters.name. The optimizee has access to the trajectory and the parameters object.

In the optimizee, three main functions have to be implemented.

1. The function create_individual() defines the individual. Here, the parameters which are going to be optimized need to be initialized and returned as a Python dictionary.
2. simulate() is the main method to invoke the simulation. The L2L framework is quite flexible about the simulation in the inner loop. It is agnostic with regards to the application carrying out the simulation and only requires that a fitness value or fitness vector is returned.
3. bounding_func() is a function that clips parameters before and after the optimization to defined ranges. For example, in an SNN, it is necessary that delays are strictly positive and greater than zero. The function is applied only on parameters that are defined in create_individual().

Similarly, the **optimizer** is created in line 18. It requires the optimizer parameters (line 17) and the method optimizee.create_individual, and if available, the bounding function optimizee.bounding_func. Additionally, a tuple of weights (fitness_weights, here (1.0, )) can be given, which weights the optimizee fitness by multiplying those values with the fitness itself. For example, in the case of a two-dimensional fitness vector, a tuple of (1.0, 0.5) would weigh the first fitness fully and the second one only half. Most of the optimizers in the L2L framework perform fitness maximization, but if minimization is required, then it suffices to flip the sign of the fitness function that would be used for maximization. Several optimization techniques are available in the framework, such as cross-entropy, genetic algorithm (GA), evolutionary strategies (Salimans et al., 2017), gradient descent, grid-search, ensemble Kalman Filter (EnKF; Iglesias et al., 2013) natural evolution strategies (Wierstra et al., 2014), parallel tempering, and simulated annealing. The results of the optimizations are automatically saved in a user specified results folder as Python binary files; however, users can store result files from within the optimizee in any format they wish.

The method run_experiment (line 24) requires that the optimizee and the optimizer and their parameters have to be defined. Finally, the end_experiment method is needed to end the simulation and to stop any logging processes.

## 3. Results

In this section, we present the results of using L2L to optimize the parameters for a variety of simulation use cases. Every task is executed with a different set of simulation tools, and the interfaces with the simulators also differ between use cases. We present here 5 use cases. Please see the Supplementary Material for an additional use case where hyper-parameters are also optimized. A GitHub repository with instructions to run the provided use cases can be found at https://github.com/Meta-optimization/L2L/tree/frontiers_submission.

### 3.1. Use Case 1: Digit Classification With NEST

**Figure 2:**
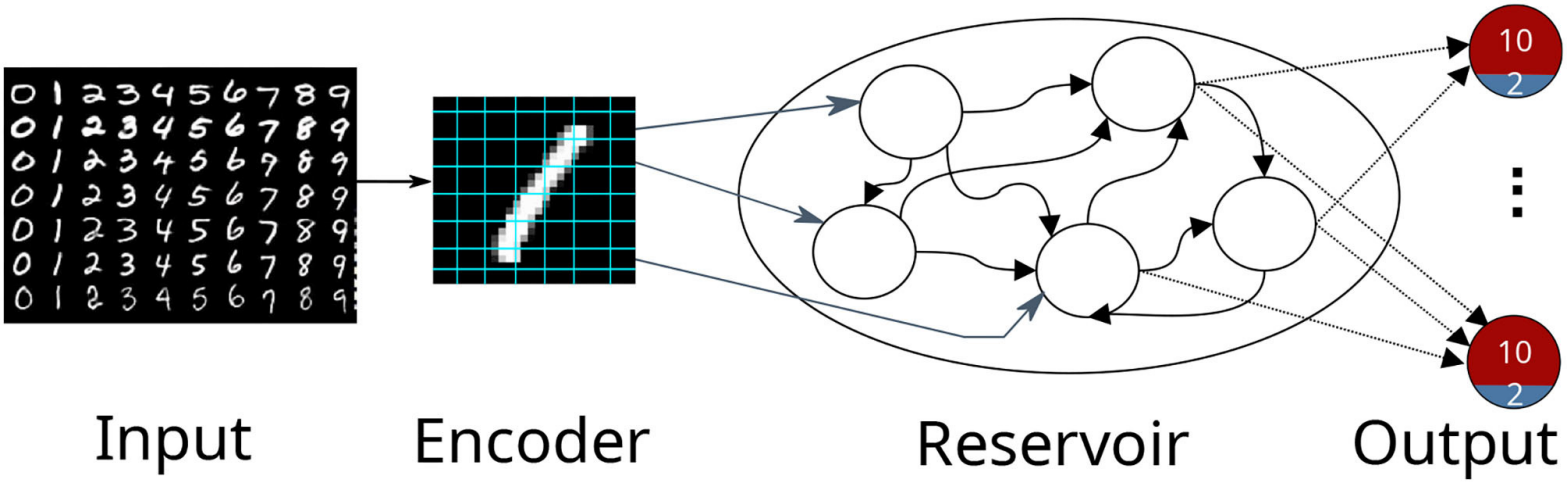
A schematic view of a reservoir network classifying the MNIST dataset. The input image is encoded into firing rates and fed afterward into the reservoir. The output consists of 10 excitatory neuron depicted in red and 2 inhibitory neurons depicted in blue. The highest activity at the output indicates the presented digit.

The first use case describes digit classification with an SNN implemented in the NEST simulator (Gewaltig and Diesmann, 2007). The SNN is designed as a reservoir, i.e., a liquid state machine (LSM, Maass et al., 2002). The network consists of an input encoding layer, a recurrent reservoir, and an output layer as shown in Figure 2. The weights between the reservoir and the output layer are optimized to maximize the classification accuracy.

#### 3.1.1. Description of the Simulation Tool

NEST is a simulator for SNN models. Its primary design focus is the efficiency and accurate simulation of point neuron models, in which the morphology of a neuron is abstracted into a single iso-potential compartment; axons and dendrites have no physical extent. Since NEST supports parallelization with MPI and multi-threading and exhibits excellent scalability, simulations can either be executed on local machines or efficiently scaled up to large scale runs on HPCs (Jordan et al., 2018). Our experiments were conducted on the HDF-ML cluster of the Jülich Supercomputing Center using NEST 3.1 (Deepu et al., 2021).

#### 3.1.2. Optimizee: Spiking Reservoir Model

The network consists of three populations of leaky integrate-and-fire (LIF) neurons, the encoder, the reservoir, and the output; see Figure 2. The input to the network is the set of MNIST digits, encoded into firing rates; the firing rates are proportional to the intensity of the pixels from 0 to 255 mapped between [1, 100] Hz. A total of 768 excitatory neurons receive input from a pixel of the image in a one-to-one connection. The reservoir has 1,600 excitatory and 400 inhibitory neurons, while the output has a population of 12 neurons (10 excitatory (red), 2 inhibitory (blue)) per digit. The connections in the reservoir are randomly connected but limited to a maximal outdegree of 6% and 8% for each excitatory and inhibitory neuron. In this setting, we focused explicitly on three digits of the dataset (0 to 2), thus having three output clusters. Each excitatory neuron receives a maximal indegree of 640 connections and each inhibitory neuron receives an indegree of maximal 460 connections from the reservoir. This results in 28, 800(= 800 × 12 × 3) connections in total. The neurons within an output are recurrently connected, while the output clusters do not have connections to each other. If an input is not presented, the network exhibits low spiking activity in all three parts. The whole network is constructed in the create_individual function. The connection weights are sampled from a normal distribution with μ = 70 and σ = 50 for the excitatory neurons and μ = −90 and σ = 50 for the inhibitory neurons.

In the simulation (simulate function), a small batch of 10 different numbers from the same digit is presented to the network for 500 ms per image as spike trains. Additionally, each neuron in the network receives background Poissonian noise with a mean firing rate of ≈5 spikes/s to always maintain a low activity within the reservoir.

Before any image is presented, there is a warming up simulation phase lasting for 100 ms in order to decay all neuron parameters to their resting values. Likewise, between every image, there is a cooling period of 200 ms where no input is shown. After the simulation is run, the output with the highest spike activity indicates the number of the presented digit.

#### 3.1.3. Fitness Metric

In the output, we acquire the firing rates of all clusters and apply the softmax function


$$\sigma {\left(\text{x}\right)}_{j}=\frac{e^{x_{j}}}{\sum_{k}e^{x_{k}}},$$

where σ :ℝ^*k*^ → [0, 1]^*k*^ and $\text{x}=\left(x_{0},x_{1},\ldots x_{k}\right)\in {\mathbb{R}}^{k}$, *j* = 1, …, *k* is the vector of firing rates.

We take the highest value, which indicates the digit the network classified. Since every image in the dataset has a label, we can calculate the loss by applying the mean squared error function to the corresponding label:


(1) $$\begin{array}{r} L=\frac{1}{n}\overset{n}{\underset{i=1}{\sum }}{\left(y_{i}-{ŷ}_{i}\right)}^{2}, \end{array}$$

with *y*_*i*_ the label and ŷ_*i*_ the predicted output, encoded as one-hot vectors with a non-zero entry corresponding to the position of the label. As the optimizer used in the outer loop for this use case is the ensemble Kalman filter, which minimizes the distance between the model output and the training label, we define the fitness function as *f* = 1 − L and use it in order to rank individuals (see next Section 3.1.4). After each presentation of a digit, the fitness and the softmax model output are sent to the optimizer.

#### 3.1.4. Optimizer: EnKF

The ensemble Kalman filter (Iglesias et al., 2013) is the optimization technique we use to update the weights between the reservoir and the output, as described in Yegenoglu et al. (2020). Before the optimization, they are normalized to be in the range of [0, 1]. The weights from the reservoir to the output are concatenated to construct one individual. In total, 98 individuals go into the optimization. Each individual has 28, 800 weights. To specify in terms of the EnKF setting, the set of ensembles are the network weights, the observations are the softmax model outputs. In Yegenoglu et al. (2020), it was shown that around 100 ensembles are required to reach at least chance level on the MNIST dataset. However, the experiments were conducted using convolutional neural networks tested with harsh conditions such as poor weight initialization and different activation functions. Due to long simulation times, we limited the number of ensembles in this case. Future work will investigate a more variable ensemble size. We implemented a slight modification of the EnKF in which poorly performing individuals can be replaced by the best individuals. The fitness is used to rank the individuals and replace the worst *n* individuals with *m* best ones. Furthermore, we add random values drawn from a normal distribution to the replacing individuals in order to increase the search space for the parameters and to find different and possibly better solutions. We set *n* and *m* to be 10% of the corresponding individuals. One hyper-parameter of the EnKF is γ (set to γ = 0.5), it can be compared to the effect of the learning rate in stochastic gradient descent. A lower γ may lead to a faster convergence but also has the risk of overshooting minima. In contrast, a higher γ is slower to converge or can get trapped in minima. Since the simulations take a relatively long time to finish, we cannot train on the whole dataset (see next Section 3.1.5) In this setting, the EnKF with the implemented additions is a suitable optimization technique, because it is able to quickly converge to minima and provide satisfactory results.

#### 3.1.5. Analysis

**Figure 3:**
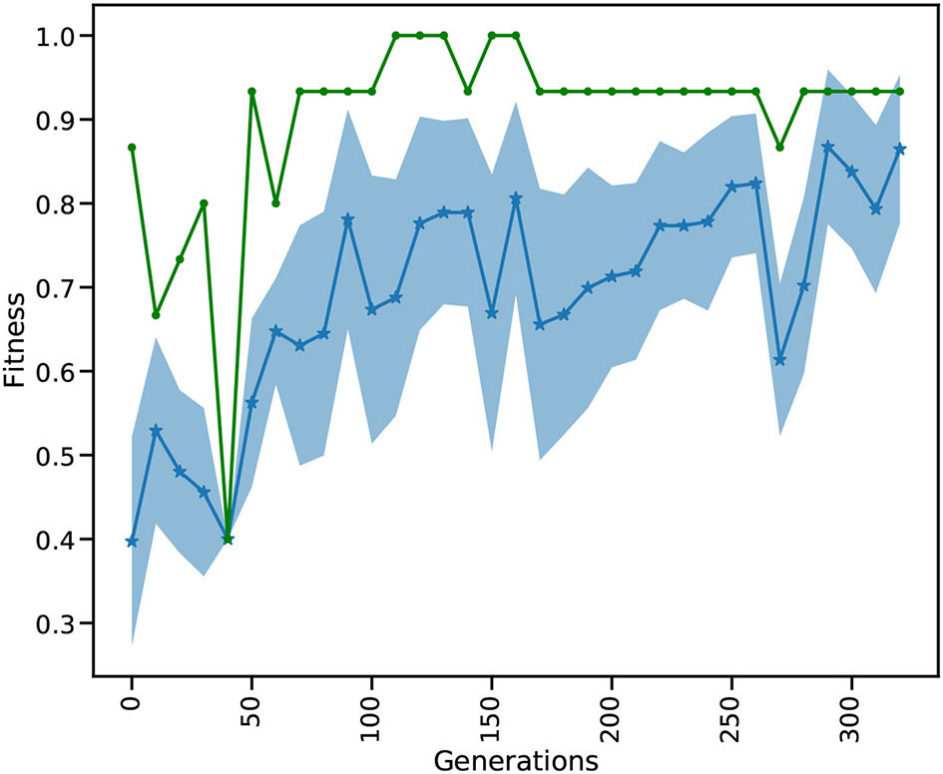
Every tenth iteration the reservoir is tested on a small part of the MNIST test data. The blue dotted line shows the mean fitness and the shaded area is the standard deviation of all individuals. The green line depicts the best fitness in every generation.

Figure 3 depicts the evolution of the fitness over 320 generations. The test is acquired over a subset of the MNIST test set in every tenth generation. The test set (10,000 images) is separated from the training set (60,000 images) and contains digits that were not presented during training.

While the mean fitness steadily increases over the generations, the best individual fitness exceeds 0.9 at generation 50 and improves to a fitness very close to 1.0 before decreasing again to around 0.9. Toward the end of training, we observe that the standard deviation of the individuals gets smaller and the mean increases. After a maximum standard deviation of 0.16 in generation 100, the spread of the ensemble contracts to a minimum standard deviation of 0.08 in generation 260, and remains low thereafter. It is important to note that the green curve indicates the performance of the highest performing individual in each generation, this is not necessarily the same individual. Currently we show 10 images for 500 ms on each generation in every training and testing phase which takes relatively long simulation times, thus hindering our ability to process the whole dataset and limits the total number of used images to 3, 200 (2, 880 training, 320 testing). Although the simulations take a relatively long time, using the HPC capabilities of L2L we are able to process an entire generation of 98 individuals including the optimization of a total of 98 ×28, 800 weights in less than 3 min. In comparison a grid search on 28, 000 parameters exploring a range of 20 values for each weight would require the evaluation of 20^28, 000^ combinations. Due to the fast convergence behavior of the EnKF it is possible to reach an optimal solution in few generations. Our modifications to sample new individuals from well performing ones and perturbing them increases the possibility to find an overall better solution by exploring other parameter ranges. A future research direction we want to investigate is to move the optimization process of the weights into the inner loop and optimize the hyper-parameters of the optimizer. In this light, it would be interesting to use Nengo or Norse which are suitable for solving ML tasks with SNNs and optimizing the hyper-parameters of the optimizers provided by those libraries. Finally, we can compare the results by executing the same approach having NEST as the SNN back-end. Our setup for learning MNIST is different from other reported works in literature in terms of architecture, learning strategy, and even metrics to measure performance. This makes a direct comparison not straightforward. Previous studies have shown a high accuracy in the MNIST dataset by shaping the structure of the reservoir. For instance, Wijesinghe et al. (2019) divide the reservoir into clusters of locally connected neurons and change the connectivity in order to reach satisfactory results on different tasks. Zhou et al. (2020) apply neural search techniques and hyper-parameter optimization using a mix of covariance matrix adaptation evolution strategy and Bayesian optimization to modify the reservoir structure, reaching an accuracy of more than 90% on the MNIST dataset. They also report high accuracy on different spatio-temporal tasks.

### 3.2. Use Case 2: Fitting Electrophysiological Data With Arbor

This use case is concerned with optimizing the parameters of a biophysically realistic single cell model implemented in Arbor such that the response of the neuron to a specific input stimulus matches an experimental recording. Both passive parameters—morphology and resistivities—and active response to an external stimulus are commonly recorded in electrophysiological experiments. Similarly, the ion channels present are typically known. However, the internal parameters of the mechanisms—usually implemented as a set of coupled linear ODEs—are not known. To address this, we use L2L to fit the model parameters to the available data. This proof-of-concept aims at providing a robust way for model fitting for the Arbor simulator using HPC resources.

#### 3.2.1. Description of the Simulation Tool

Arbor is a library for writing high-performance distributed simulations of networks of spiking neuron with detailed morphologies (Akar et al., 2019). Arbor implements a modification of the cable-equation model of neural dynamics which describes the evolution of the membrane potential over time, given the trans-membrane currents. In this model, neurons comprise a tree of *cables* (the morphology), a set of dynamics assigned to sub-sections of the morphology (called *ion-channels* or *mechanisms*), and a similar assignment of bio-physical parameters. The morphology describes the electric connectivity in the cell's dendrite and the mechanisms primarily produce the trans-membrane currents.

#### 3.2.2. Optimizee: Morphologically-Detailed Single Cell

As outlined above, we expect models to be imported from laboratory data, that is a morphological description of the cell from microscopy, a template of ion channels with yet unknown parameter values, and some known data like the temperature of the sample. In addition, a series of stimulus and response measurements need to be provided, which will be the target of optimization. Our objective then is to assign values to the parameters to best approximate the measured response. For designing this use case, we focus on a single specimen from the Allen Cell Database with a known parametrization in addition to the input/response data (Lein et al., 2007).

We define the parameter sets **P** to be fit as a list of 4-tuples: a sub-section of the morphology, an ion-channel id, a parameter name, and the value to set the parameter to. Regions in the morphology are written as queries against Arbor's layout engine, e.g., selecting all parts of the dendrite where the cable radius is smaller than 1 μm becomes (rad-lt (tag 2) 1), since tag=2 has been set during morphology creation. Consequently, setting the parameter tau in the expsyn mechanism to 2 ms appears as

[.., ((rad-lt (tag 2) 1), expsyn,
tau, 2), ..]

in the individual. Optimizee instances are constructed from are configuration file which lists the following items (example item)

- morphology file name (cell.swc)
- list of current clamps with expected response (delay, duration, amplitude, ref.csv)
- simulation parameters: length and time-step
- location where to record the response (location 0 0.5)
- fixed parameter assignments (T=285 K)
- list of ion channel assignments and optimizable parameters [(tag 2), pas, e, -70, -30]

Parameters to be optimized are given a bounding range used to automatically restrict the optimizer, here e may vary in the range of [-70 mV… -30 mV]. This data is sufficient—together with the statically known items—to construct a simulation in Arbor that can be run forward in time.

#### 3.2.3. Fitness Metric

We implemented the naive approach of using the mean square loss as the measure of fitness. Given the experimentally obtained membrane potential *U*_ref_(*t*) we define the fitness as


(2) $$\begin{array}{r} L\left(\text{P}\right)=-\frac{1}{T^{2}}\overset{T}{\underset{t=0}{\sum }}{\left[U_{\text{ref}}\left(t\cdot \tau \right)-U_{\text{sim}}\left(\text{P},t\cdot \tau \right)\right]}^{2} \end{array}$$

where *U*_sim_(**P**, *t*) is the measurement produced by Arbor given the parameter set **P** and τ is the sampling interval of the voltage measurement. The optimizer attempts to maximize the given metric, which is why we defined the fitness as the negative of the *L*_2_ norm here.

**Figure 4:**
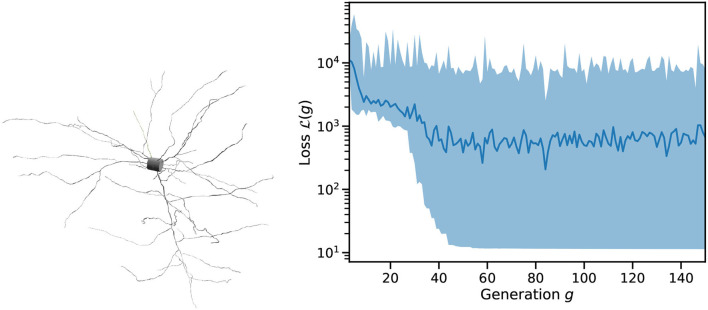
Example input to Arbor and trace of a run of the optimizer. **(Left)** Cell morphology as consumed by Arbor and imported from the Allen DB, regions are marked as “soma,” “dendrite,” and “axon.” **(Right)** Loss function over successive generations of the genetic optimizer for an example run of L2L on this cell starting from random parameters. Shown are the mean loss per generation (as a line) and the spread between minimum and maximum (shaded area).

**Figure 5:**
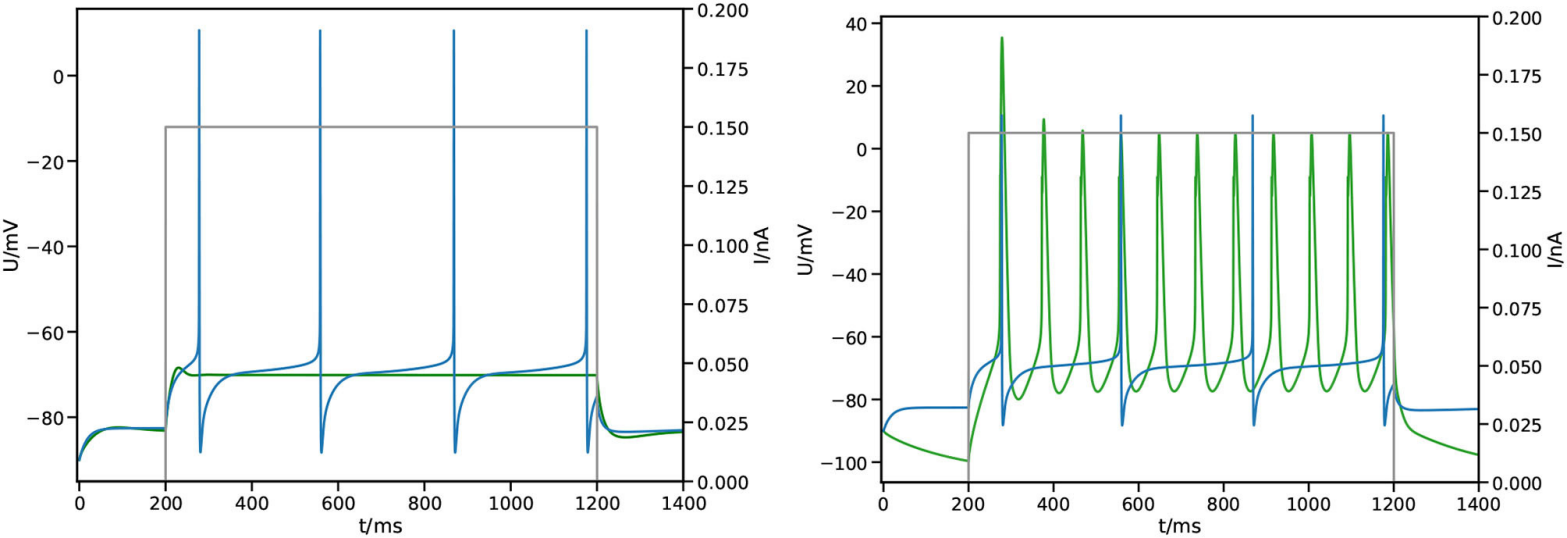
Impact of the fitness function. Shown is the measured membrane potential at the center of the soma from the simulation 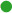 against reference 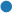 and applied stimulus 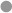. **(Left)** Simple square loss, the best result after 100 generations. **(Right)** Feature based fitness, the best result after 100 generations.

Figure 4 shows an example of single cell morphology and the loss function across a single run of L2L. The scales and units of L are arbitrary. After roughly 50 generations, the best result has been identified and we found only minor improvements to the fitness after this. As can be seen in Figure 5 (left), we quite easily reach a configuration that reproduces the *mean* membrane voltage but does not exhibit spiking behavior. From experience, we know that spikes are only produced for a narrow band of parameters in these complex configurations.

Thus, the fitness function will need to be extended to include the requirement for spiking. Furthermore, it seems prudent that the final result of the optimization process should include the responses to multiple separate stimulation protocols. Therefore, the overall fitness becomes a vector


(3) $$\begin{array}{r} F\left(\text{P},I\right)=\left(\begin{array}{c} L\left(\text{P},I_{0}\right)\\ S\left(\text{P},I_{0}\right)\\ L\left(\text{P},I_{1}\right)\\ \vdots \end{array}\right) \end{array}$$

which—in conjunction with a vector of weights—is suited for use with L2L's multi-objective optimization. Here, **I** is the vector of stimuli and the function S collects the fitness with respect to the spiking behavior. Thus, the fitness function was changed to emphasize spiking behavior


(4) $$\begin{array}{rc} L\left(\text{P},I\right) & =|\langle U_{\text{ref}}\rangle -\langle U_{\text{sim}}\rangle | \end{array}$$

(5) $$\begin{array}{rc} S\left(\text{P},I\right) & =\langle U_{\text{ref}}-U_{\text{sim}}\rangle {}_{U_{\text{ref}}>\sigma } \end{array}$$

where S selects spikes by applying a threshold σ and then applies the temporal average 〈·〉. As can be seen in Figure 5 (right), we find spiking behavior with this fitness function, albeit still different from the expected outcome.

#### 3.2.4. Optimizer: Evolutionary Algorithm

The fitness metric is used to drive the outer loop optimizer, an evolutionary algorithm searching for maximum fitness. This choice of the algorithm was motivated by prior studies showing it to be computationally efficient for this kind of fitting problem (Druckmann et al., 2007).

In the L2L framework, the genetic algorithm optimizer (GA) is a wrapper around the DEAP library (Fortin et al., 2012). This adapter takes care of handling the parameters received from the inner loop and prepares them for the optimization process. The DEAP library then facilitates the cross-over and mutation methods, applies them to the actual parameter set, and saves the best individuals into the Hall of Fame if they fare better than previous runs. Afterward, the optimized parameters are sent back to the optimizee, which then initializes the next generation of individuals.

Here, we use a population of 100 individuals and a total of 200 generations. Individuals in a generation are evaluated by using 16 parallel tasks on a single dual-socket node.

#### 3.2.5. Analysis

We have shown a basic implementation for finding optimal parameter sets for single cell models using Arbor and L2L. This enables researchers to fit experimental data to neuron models in Arbor, a workflow that is important in practice and lacking so far in Arbor's ecosystem. The approach shown here so far is implemented in a straightforward fashion but falls short to reach the desired configuration in a reasonable time frame.

A fitness function based on salient features is generally more successful in producing spiking behavior (Druckmann et al., 2007; Gouwens et al., 2018). We expect the current fitness implementation to evolve further, likely including more features, such as the resting potential and mean spike frequency. Further, L2L does not normalize parameters, thus parameters that have significantly different ranges can pose issues to the optimization process, e.g., the test case here features parameters of magnitude 100 as well as 10^−7^. Given the bounding annotations in our configuration, we implemented normalization within the optimizee and L2L handles uniform ranges [0, 1]^4^. To cope with common time-restrictions on the used resources in the mean-time, we implemented a method to resume optimization given an intermediate result. Currently, this workflow is being extended beyond the proof-of-concept state we presented here. A further open task is to investigate the impact of the hyper-parameters passed through L2L to DEAP, such as tournament size, population size, etc.

Another extension is the use of accelerators (GPUs), which allow for massively parallel evaluation of individuals. Arbor is able to use GPUs for simulations efficiently starting at a few thousands of cells per GPU. This would enable processing an entire generation of the optimization process at once. Given the current number of 100 cells per generation, this is not yet profitable, but for larger generation sizes and additional stimulus protocols, it becomes attractive. L2L was extended to enable a vectorized version of the evolutionary algorithm similar to the multi-gradient descent approach presented in use case 4 (Section 3.4).

### 3.3. Use Case 3: Foraging Behavior With Netlogo and NEST or SpikingLab

**Figure 6:**
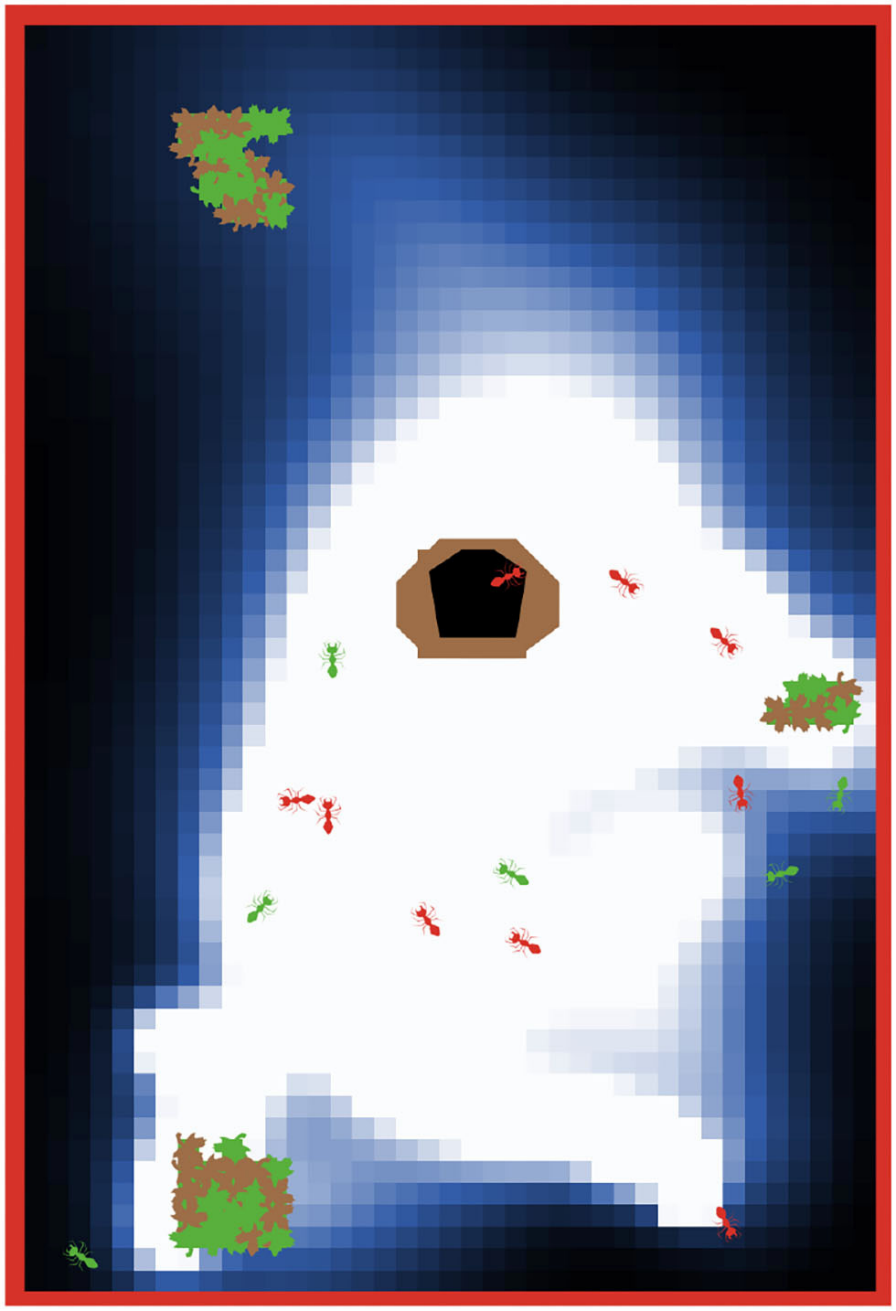
The ant colony is searching for food (big green patches with brown leaves). The ants are communicating *via* pheromones which are dropped on the ground (blue-white patches) when food is found or when the ants return to the nest (black-brown patch). Green colored ants are transporting the food, while orange colored ants are exploring the environment or following the pheromone trail. The red border around the world is an impenetrable wall and prevents ants from crossing from one side to the other. The pheromone trail decays with time if it is not reinforced by other ants.

In this use case, we describe optimizing the foraging behavior in a simulated ant colony. The colony consists of 15 ants, all of which are searching for food (big green patches, Figure 6). Any food found must be brought back to the nest. Ants communicate with each other by dropping pheromones on the ground (blue patches) whenever the food is found or the nest is reached. The pheromone can be smelled by other ants which then can follow the trail left on the ground. Each ant is controlled by an SNN, which is an identical copy for every ant. Here, we use L2L to configure its weights and delays so that the ants bring food back to the nest as efficiently as possible.

#### 3.3.1. Description of the Simulation Tools

NetLogo is a multi-agent simulator and modeling environment (Tisue and Wilensky, 2004). It is widely used as an educational and scientific tool for the study of emergent behavior in complex systems. Agents are expressed as objects that can communicate with each other. In our setting, NetLogo helps us to observe and manipulate the state of every neuron and synapse. For the simulations, we have two backends: NEST (see Section 3.1.1) and SpikingLab (Jimenez-Romero and Johnson, 2017). SpikingLab is an engine directly integrated within NetLogo and can be easily and quickly used for small scale networks, as we present in our use case. Invoking NEST from NetLogo causes a minimal communication overhead since NEST needs to be called as an external process. For larger networks, it is preferable to use NEST since its higher simulation efficiency compensates for the communication overhead.

#### 3.3.2. Optimizee: Simulated Ant Brain

**Figure 7:**
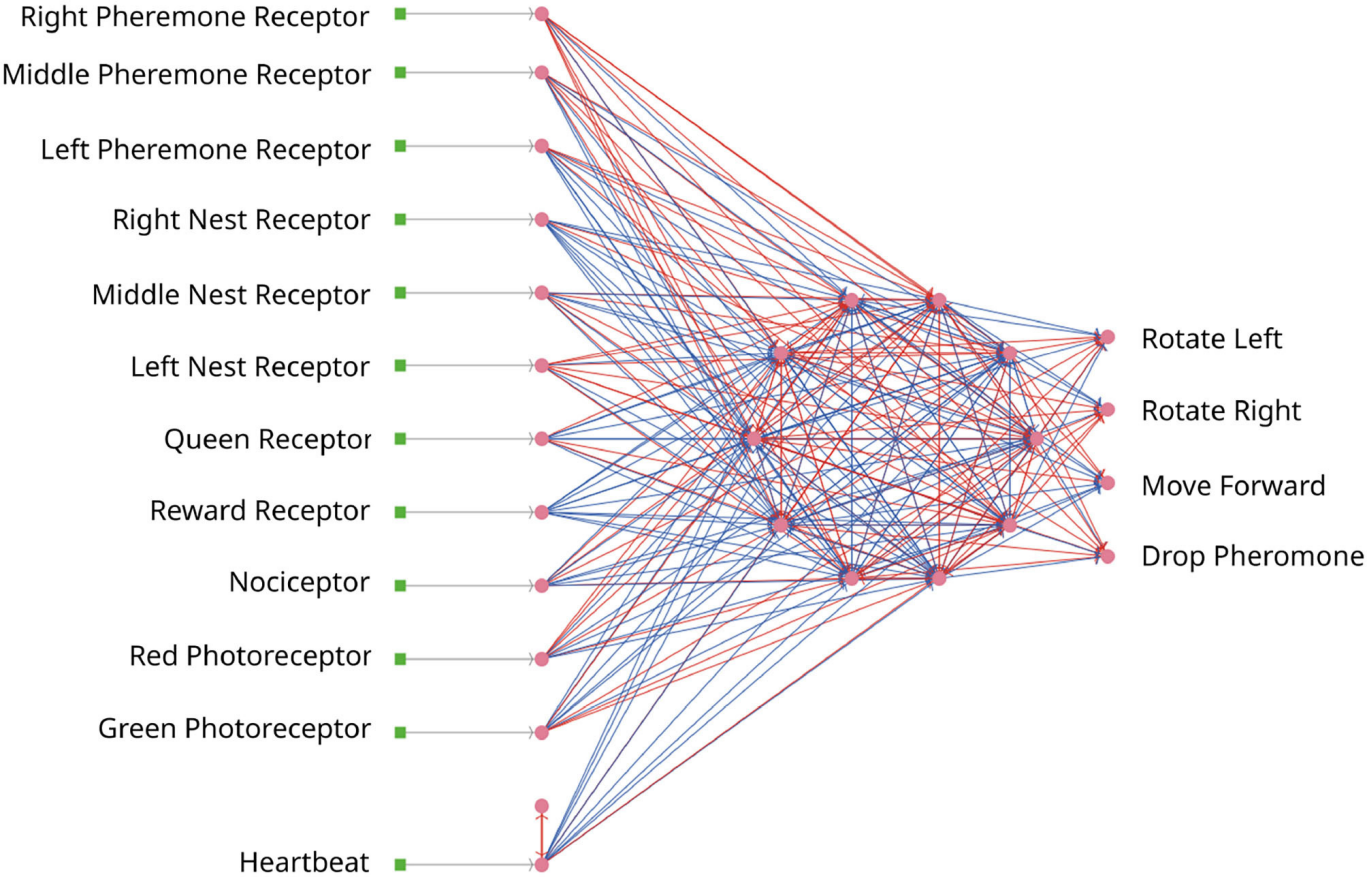
The SNN for the ant colony. Every ant is steered by an SNN. Neurons are depicted as pink dots and excitatory/inhibitory connections as red/blue lines. All networks are identical.

In the first iteration, the optimizee creates the individual inside the create_individual function. The individual consists of network weights and delays. The weights are uniformly distributed in [−20, 20], while the delays range between ${\left[1,\ldots ,7\right)}_{{\mathbb{N}}^{+}}$. The network has an input, a hidden, and an output layer, the neurons are all-to-all connected for every layer as depicted in Figure 7. The input layer consists of 12 neurons. The first three neurons are receptors to smell the direction of the pheromone. The next three neurons are responsible to locate the nest. The queen receptor indicates the middle of the nest. Reward and nociceptors determine the reward and punishment for the ant. The green and red photoreceptors are triggered when food or a wall is seen. Finally, the heartbeat neuron stimulates the network in every timestep with a small direct current to keep a low dynamic ongoing in the network. The four output neurons are responsible for the movement and for dropping the pheromone. Similar to the first use case in Section 3.1.5, the total number of individuals is 98. The total number of connection weights (250) and delays (250) is derived as follows: 110 connections from the input to the middle layer, 10 connections from the heartbeat neuron to the middle layer, 90 connections in the middle layer, and 40 connections from the middle layer to the output (110+10+90+40 = 250). The weights and delays can be min-max normalized if specified. The optimizee saves these parameters as a csv file before starting the simulation. The model is invoked by a Python subprocess^5^ in the simulate function, which then calls the headless mode of NetLogo to start the run. The optimizee waits until the simulation is finished and collects the fitness value from a resulting csv file which is written after the simulation ends.

The user has to set whether NEST or SpikingLab is invoked as a backend inside the simulation. NEST is known as a subprocess by NetLogo, while SpikingLab is directly accessed by the model. In the case that NEST is selected, the parameters have to be passed to it as well since the network needs to be constructed with the new parameters. This can be done either by loading the parameter in a csv file within NEST, or NetLogo can read the csv file and pass the values to the simulation.

The parameters are restricted within the bounding_func function if their values exceed the specified ranges after the optimization process. Weights are clipped to the range of [−20, 20] and delays to [1, 5].

#### 3.3.3. Fitness Metric

The fitness function for the ant colony optimization problem rewards finding food and bringing it back to the nest while punishing excessive movement.

We define the ant colony fitness *f*_*i*_ of optimizee *i* as:

(6) $$\begin{array}{r} f_{i}=\overset{T}{\underset{t=1}{\sum }}\left(\overset{J}{\underset{j=1}{\sum }}N_{i,j}^{\left(t\right)}+F_{i,j}^{\left(t\right)}-C_{i,j}^{\left(t\right)}\right), \end{array}$$

where *t* = 1, …, *T* is the simulation step, *T* is the total simulation time, *J* is the total number of ants in the colony, and *j* indexes the ants. N is the reward for coming back to the nest with food, F is a reward for touching the food, and C is the movement cost. Every movement, rotation, and pheromone dropping is added toward C. We set the cost as follows: Rotation −0.02, pheromone dropping −0.05, and movement −0.25. The movement has a higher cost since we would like to restrict vast movements and force them to return to the nest. We also punished resting with −0.5 to speed up the movement and to slightly induce exploration. The rewards are returning to the nest 220 and touching food 1.5. A high reward for coming back to the nest is necessary, otherwise, the ants are spending a long time exploring the environment even when the food is found. This slows down learning and hinders solving the task.

#### 3.3.4. Optimizer: Genetic Algorithm

We use a genetic algorithm to optimize the weights and delays in the ant brain network. This is the same class of optimizers as used in Section 3.2.

#### 3.3.5. Analysis

**Figure 8:**
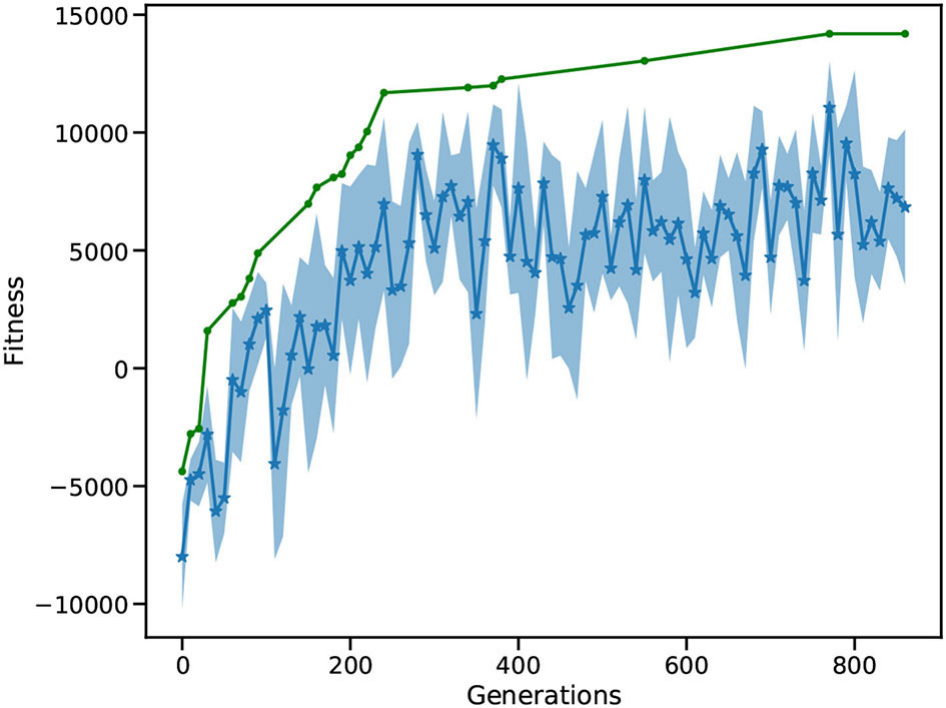
Fitness of the ant colony. The blue curve shows the mean fitness and the shaded area is the SD. The green curve indicates the best solution found so far, and thus rises monotonically.

Figure 8 depicts the evolution of the fitness of the ant colony over 800 generations. Initially, the ants move a lot without retrieving food, resulting in a negative maximum fitness. After around 200 generations, the mean fitness is consistently positive and the best solution is close to 10,000. In following generations, the mean fitness saturates at around 5,000, with increasing best fitness. After 800 generations, the L2L run is stopped with the best individual fitness close to 15,000. Similarly to use case 3.1, L2L enables us to execute 98 individuals in parallel, where a generation is optimized in less than 2 min. A grid search algorithm with 20 values to explore weight and delay combinations would require 20^500^ possibilities to test for. The mutation and cross-over steps of the GA increase the parameter space and avoid local minima, without loosing performance. The best individuals are saved in the Hall of Fame (HoF) if they have better fitness than their predecessors. If an optimization step produces underperforming individuals, it is possible to recombine the new set utilizing the HoF. Due to the parallel distribution of individuals and the GA optimizer, we are able to find well performing individuals in less than 400 generations. In contrast to other literature optimizing ant colonies using rule-based systems, our work describes the optimization of an SNN that learns the foraging behavior of an ant. The decision making of each ant is not based on fixed rules (e.g., if food is found turn around 180° and go back to the nest), instead, it depends on the firing activity of the network in response to the perceived environment. Compared to the ant colony model provided by NetLogo (Wilensky, 1997), which solves the foraging task within ≈15,000 steps, our SNN solution takes between 15,000 and 20,000 steps with a diffusion rate of 20 and evaporation rate of 1. However, when the environmental conditions change to the detriment of the pheromone communication (e.g., the evaporation rate increases and diffusion rate decreases), the performance of the two implementations becomes closer. In general, utilizing the network solution enables the ants to be more adaptable toward environmental modifications.

### 3.4. Use Case 4: Fitting Functional Connectivity With TVB

This use case describes tuning the parameters of a whole brain simulation using the GPU models of The Virtual Brain simulator (TVB; Sanz Leon et al., 2013) to give the best match to empirical structural data.

To do clinical research with TVB, it is often necessary to configure the parameters of a model for a specific person such that it matches obtained empirical data. First, the brain is parcellated into different regions, based on many available atlases (Bansal et al., 2018). The connectivity of these regions is determined using diffusion weighted imaging, estimating the density of white matter tracts between the regions, resulting in a connectivity matrix which is regarded as the structural connectivity. Finally, a model that represents the regional brain activity must be chosen. To optimize the match between a specific person and the TVB simulation, obtained fMRI can be used to further personalize the structural connectivity (Deco et al., 2014).

Due to the high dimensionality of TVB models and the wide variation in possible parameter values, fitting patient data often requires extensive parameter explorations over large ranges. In this use case, the simulated functional connectivity is matched to the structural connectivity. The task has the underlying assumption that regions that are anatomically connected often show a functional connection (Honey et al., 2009). In this task, we want to find the values for the global_coupling and global_speed variables, characteristic of the connectome of a TVB stimulation, which gives rise to the strongest correlation between the structure of the brain and the functional connectivity, i.e., the relationship between spatially separated brain regions.

#### 3.4.1. Description of the Simulation Tools

The Virtual Brain is a simulation tool which enables researchers to capture brain activity at mesoscopic level using different modalities such as EEG, MEG of fMRI, using realistic biological connectivity. A TVB brain network consists of coupled neural mass models (NMM) whose dynamics can be expressed by a single or system of ordinary differential equations. The coupling of the NMMs is defined by the connectivity matrix. The NMMs describe, e.g., the membrane potential or firing rate of groups of neurons using differential equations, which are then solved numerically. In this use case, we utilize an Euler based solver. RateML (van der Vlag et al., 2022), the model generator of TVB, enables us to create the desired TVB model written in CUDA for the GPU and a driver to simulate the model, from a high level model XML file.

**Figure 9:**
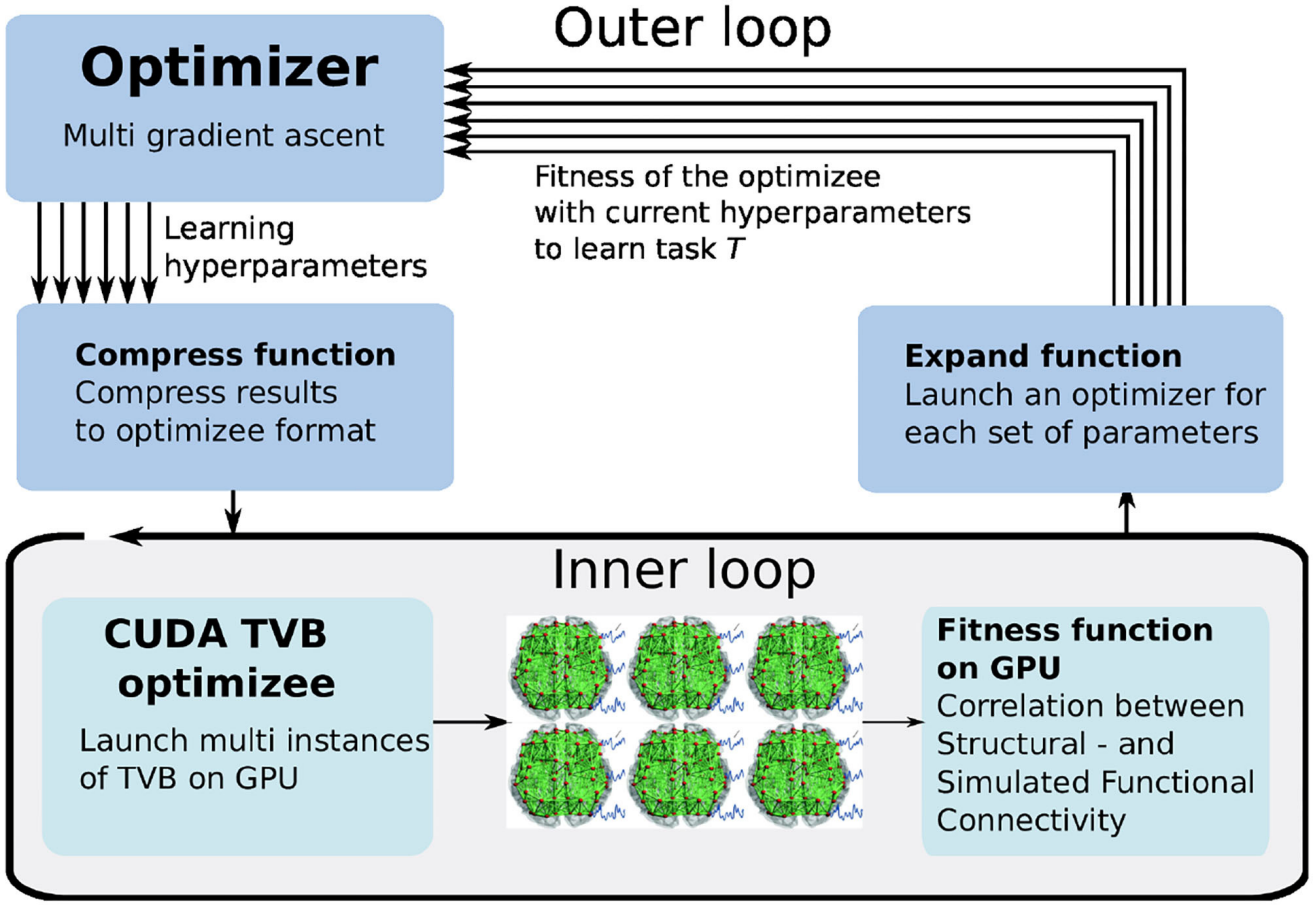
The multi-gradient ascent implementation of L2L. The inner loop launches multiple instances of TVB on the GPU simulating different sets of parameters. The outer loop selects the best fitnesses and produces a new parameters range.

Unlike the use cases discussed above, in this case, we exploit GPU-parallelization by defining an optimizer that can process a vector of fitnesses and create new individuals for multiple TVB simulations executed in parallel on the GPU. An overview of this process is shown in Figure 9. The optimizee in the inner loop spawns a number of threads (here: 1, 024) according to the users defined parameters ranges and resolution. Each thread represents a TVB instance, simulating a unique set of parameters. The fitness is computed for each instance, and the outer loop optimizer selects the best fitness by using the gradient ascent strategy. The arrows indicate the independent iterations of the vector of fitnesses. In the figure, six TVB simulations run in parallel, thus the optimizer needs to iterate a vector of six fitnesses.

#### 3.4.2. Optimizee: Whole Brain Simulation

The create_individual function initializes a first instance for the TVB simulation. The structural connectivity is usually obtained from the patient but in this case, the standard TVB connectivity for 76 nodes is used. We model the regions with the Generic2DimensionOscillator (G2DO; Ott and Antonsen, 2008). A dictionary is created which contains initial random values for the optimization parameters, connection_speed and coupling_strength.

For subsequent simulation generations, the optimizee reads the adapted values from a text file written by the optimizer and utilizes the Python subprocess module to spawn a new TVB simulator object with the corresponding parameterization. When the TVB simulation is complete, the fitness for each TVB instance is computed and written to a separate text file. The text files are read by the optimizee reformatted for processing by the optimizer.

#### 3.4.3. Fitness Metric

The computation of the fitness for this task is 2-fold. In the first step, the simulated functional connectivity is determined by computing the Pearson product-moment correlation coefficient, ρ_*xy*_, of the simulated 76 regions according to Equation 7.


(7) $$\begin{array}{r} \rho_{xy}=\frac{\text{Cov}\left(x,y\right)}{\sigma_{x}\sigma_{y}}, \end{array}$$

where Cov(*x, y*) is the covariance of variables *x* and *y* and σ_*x*_ and σ_*y*_ are the SD. This first step determines how strong the dynamics of the simulated regions correspond to one another. A strong functional correlation means that the simulated activity between the spatially separated brain regions is more similar. The second step is to determine the correlation between the obtained functional and the structural connectivity, the weight matrix used in the simulation, also using Equation 7. The Python implementation of the second step is shown in Listing 2, where SC is the structural connectivity and FC is the simulated functional connectivity that was computed previously. On line 1, the weights are normalized. In the for-loop on line 2, the correlation with the structural connectivity is computed. The FCSC holds these correlations and is the array of fitnesses returned to the optimizer.

**Listing 2:**
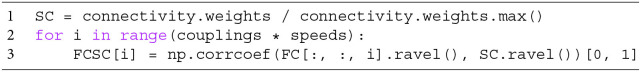
Implementation of the correlation computation between functional and structural connectivity.

#### 3.4.4. Optimizer: Multi-Gradient Ascent

The best fitness is selected with a gradient ascent optimizer. The existing optimizer has been adapted for processing the vector of fitnesses returned by the GPU, named multi-gradient ascent (MGA). In order to adapt it to vector processing, the fitnesses need to be expanded before processing and compressed afterward, as is shown in Figure 9. The expansion transforms the obtained fitnesses from the optimizee process to a data structure in which the obtained fitnesses are linked to the used parameters, thus enabling the multi-gradient ascent optimizer the possibility to select the best fitness and define a range for the new parameters to be sent to the optimizee. When the optimizer has selected the parameters for the optimizee, it compresses the new individuals to a data structure that just contains the new parameter combinations for the optimizee. Aside from the expanding and compressing, the MGA algorithm determines the new values for the individuals similar to gradient ascent.

#### 3.4.5. Analysis

**Figure 10:**
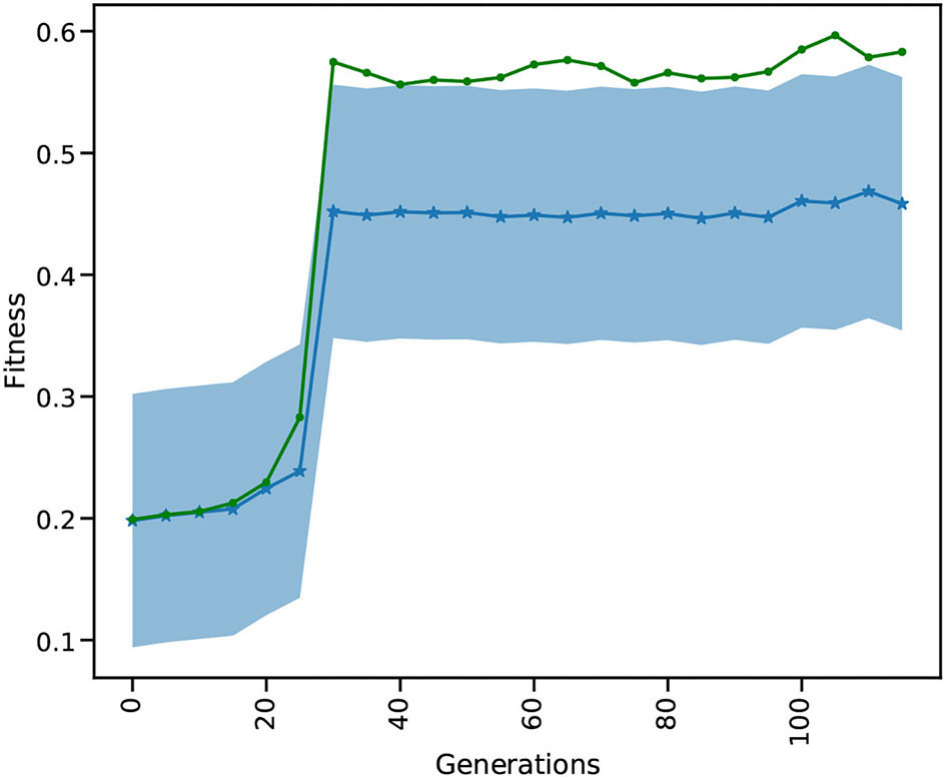
SD of the mean and best out of 1,024 fitnesses for 116 generations for the multi-gradient TVB parameter optimization TVB simulation. The blue curve is the mean fitness over the population of 1,024 and the shaded area gives the SD. The green curve shows the best fitness for each generation.

The results in Figure 10 show the evolution of the mean and best fitness for a generation of 1, 024 parameter combinations for the global_speed and global_coupling variables, with a learning rate of 0.01 and four individuals. These four individuals each spawn 1,024 TVB simulations on the GPU, enlarging the chance of success. Each generation contains a TVB simulation of 4,000 simulation steps with a *dt* = 0.1. These results were obtained using a NVIDIA V100 GPU on the JUSUF^6^ cluster. Our results show that after 30 generations the best attainable fitness (green curve) is reached (c.f. Deco et al., 2014).

Comparing the GPU population based on a single L2L implementation, the latter would need more generations before the best fitness is attained. The likelihood of finding a suitable solution in earlier generations rises with the size of the population: the more configurations considered in a single generation, the faster it converges to the best value. The GPU implementation has already considered 30 × 1,024 different parameters values, after which the optimal fitness is found (Figure 10), while the single implementation would have only 30. A single implementation would need at least 30,720 generations to find the same result, but would very likely need many more. Additionally, the GPU makes it very convenient to execute many simulations in parallel by not having to split them up onto multiple nodes, without communication overhead and decreasing wall clock time even further.

### 3.5. Use Case 5: Solving the Mountain Car Task With OpenAI Gym and NEST

**Figure 11:**
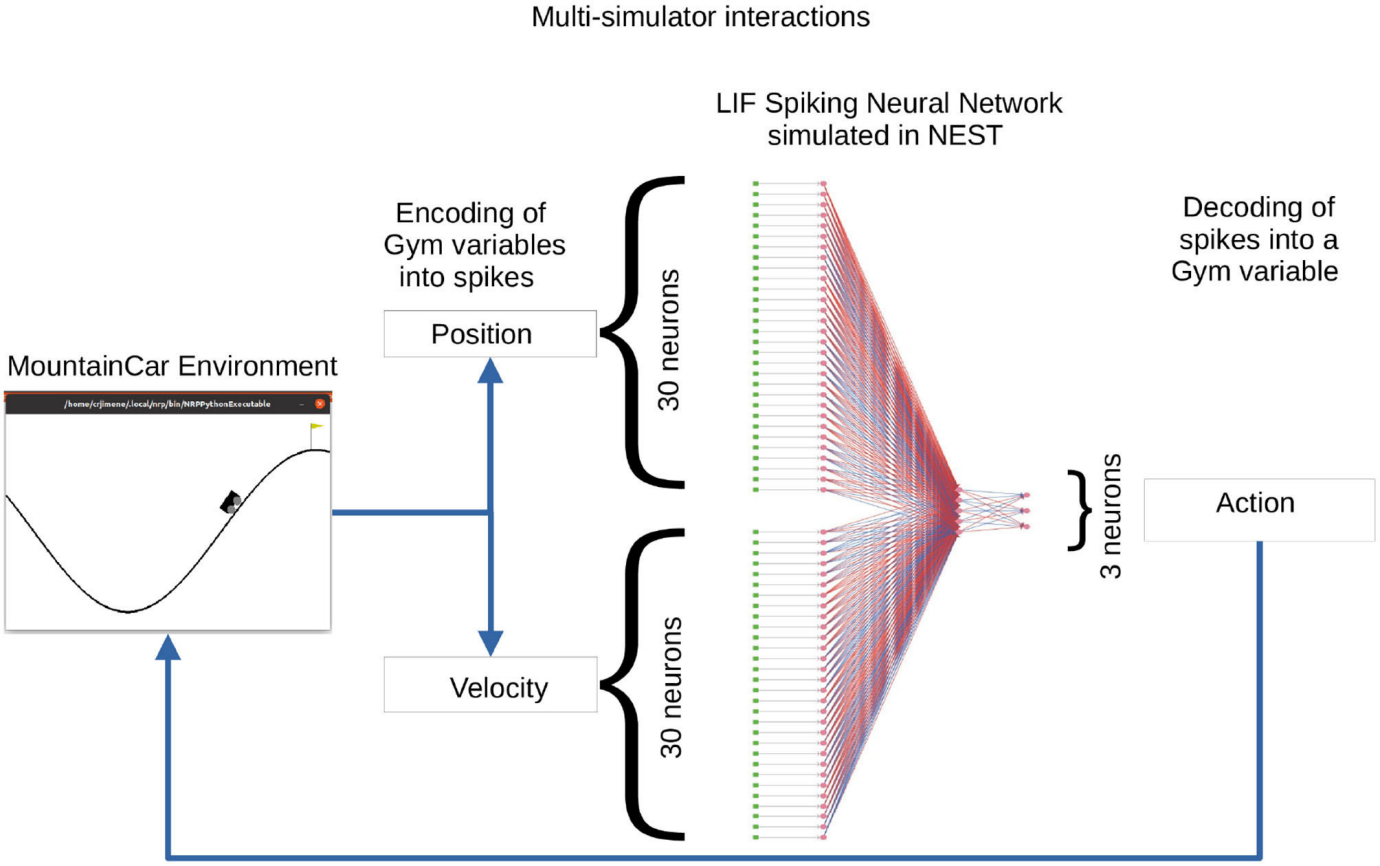
A feed-forward spiking network to solve the Mountain Car task. In the Mountain Car environment (left) the agent must steer the car to reach the goal position (flag on top of the hill). The position and velocity of the car reported by the MC are encoded into spikes by the encoding layer (DC generators depicted as green dots) of the three-layer spiking neural network (SNN) (neurons depicted as pink dots and excitatory/inhibitory connections as red/blue lines) running in the NEST simulator (right). The activity from the three output neurons (accelerate left, accelerate right, do nothing) is decoded into actions for MC. The set of weights between the sixty encoding neurons and the three output neurons is the object of optimization.

In this use case, we describe a solution to the OpenAI Gym Mountain Car (MC) problem. The MC task is interesting since it requires the agent to find a policy in a continuous state space constituted by the position and velocity of the car. At the same time, the action space is discrete, limited to three possible actions: accelerate left, accelerate right, and do nothing. The initial position and velocity of the car are set randomly by the environment; the aim is to reach the goal position (yellow flag) as depicted in Figure 11. As the car's motor is weak, consistently reaching the goal at the top of the hill requires the agent to learn a policy that swings the car back and forth in order to build up momentum. The challenge is considered solved if the car reaches the goal position in an average of 110 steps over 100 consecutive trials. We implement a feed-forward LIF SNN in NEST to encode a policy and optimize the weights so as to improve the ability of the network to solve the task.

#### 3.5.1. Description of the Simulation Tools

The OpenAI Gym (Brockman et al., 2016) is a software library that provides an interface to a wide range of environments for experimentation with reinforcement learning techniques. NEST has been described in Section 3.1. Both simulators are instantiated and invoked by the optimizee process which implements the closed-loop interactions. These interactions are synchronized in such a way that for each simulation step of the MC environment, the SNN is simulated for an interval of 20 ms in NEST. On completion of a simulation interval, the state of the network is sampled and fed back as an action to the MC environment.

#### 3.5.2. Optimizee: Spiking Feed-Forward Policy Network

The SNN of LIF neurons that controls the actions of the car is implemented in NEST. The inputs to the SNN are the position [−1.2, 0.6] and velocity [-0.7, 0.7] variables which are discretized and encoded using 30 input neurons for each variable. For the discretization (binning) of the continuous variables, the width (*w*) of the bins is given by the minimum (*min*) and maximum (*max*) value of the interval divided by the number of input neurons (*n*) available for each variable. Each value within the range is discretized into a bin which corresponds to one input neuron:

$$\begin{array}{r} w=\frac{min+max}{n}\text{(8)} \end{array}$$

Once a value falls into a bin, its corresponding neuron is activated by a dc current as provided by a connected dc generator resulting in a firing rate of 500 Hz. The 60 encoding neurons have all-to-all connections to an intermediate layer of five neurons, which in turn have all-to-all connections to the three neurons in the output layer corresponding to the three possible actions. The action sent to the OpenAI Gym environment depends on the activity of the three neurons in the output (third) layer. Each output neuron represents one of the possible actions. Following a winner-takes-all approach, the neuron with the highest spiking activity determines which action is sent to the OpenAI Gym environment. Figure 11 illustrates the spiking network and the closed-loop interaction with the MC environment on the basis of input variables and output actions.

Similar to the Netlogo use case (see Section 3.3), at the beginning, the optimizee creates the individual inside the create_individual() function. The total number of individuals per generation is 32. Each individual consists of network weights, which are initially uniformly distributed in [−20, 20]. There are 315 weights corresponding to the (60 × 5) + (5 × 3) = 315 synaptic connections in the network. The instantiation and orchestration of OpenAI Gym and NEST simulator (including the set-up of the SNN) is carried out by the optimizee. Each simulation runs for 110 simulation steps (where a simulation step corresponds to an action being sent to the environment) or until the goal position is reached. Once the simulation is completed, the optimizee returns the calculated fitness value to the optimizer. The bounding_func() function ensures the weights are clipped to the range [−20, 20] if the values exceed this range after the optimization process.

#### 3.5.3. Fitness Metric

The fitness function for the MC optimization problem is defined as the maximum horizontal position reached by car during an episode comprised of 110 simulation steps, i.e.,


$$\begin{array}{l} f={\text{max}}_{T}({\vec{P}}_{T}) \end{array}$$

Where max_*T*_ returns the item with the highest value in a vector and ${\vec{P}}_{T}$ contains the position of the car on each simulation step up to *T* = 110.

#### 3.5.4. Optimizer: Genetic Algorithm

The optimization method is identical to that used in Section 3.3. Afterward, the optimized parameters are sent back to the optimizee, which then initializes the next generation of individuals.

#### 3.5.5. Analysis

**Figure 12:**
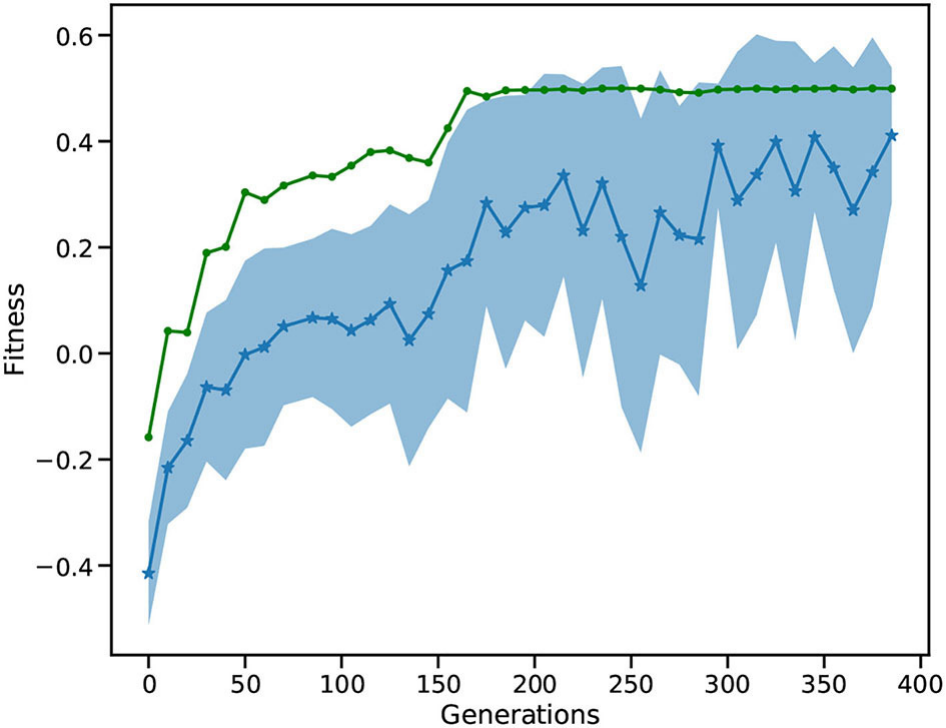
Fitness of the MC run. The blue, starred line shows the mean of all individuals while the shaded area is the SD. The green line is the best fitness per generation.

Figure 12 depicts the fitness of the SNN over 400 generations. After 50 generations, the fitness becomes positive showing that the car is moving toward the goal position. The best solution (goal position of 0.5) is first reached around generation 160. In following generations, the mean fitness saturates at around 0.3, while the best fitness reaches the maximum of 0.5. After 400 generations, the L2L run is stopped with the best individual fitness being 0.5. Finally, we confirmed that the fittest individual could solve the MC problem. We ran a thousand episodes (each episode lasting for a maximum of 200 simulation steps); the spiking network achieved the required average of 110 or less simulation steps over 100 episodes. Our solution requires 101 simulation steps on average to reach the goal position and thus solves the task (data not shown).

The Mountain Car problem has been approached using several ML techniques most of them focusing on reinforcement learning (Heidrich-Meisner and Igel, 2008; Weidel et al., 2021) and gradient descent (Young et al., 2019). Current implementations are able to solve the challenge while delivering a good performance in terms of speed of convergence and the obtained final score. We took an evolutionary approach by using a GA to optimize an SNN that is able to solve the MC obtaining consistently a high reward (over 100 trials). Evolutionary strategies have shown comparable performance to reinforcement learning and gradient descent algorithms in problems where learning to sense and act in response to the environment are required (Salimans et al., 2017; Such et al., 2017; Stanley et al., 2019). Another advantage with the evolutionary approach is the parallel exploration of the solution space. In L2L, each individual is run as an independent optimizee process. The framework enables us to execute a large number of parallel optimizees in multi-core CPUs and HPC infrastructures.

## 4. Discussion and Future Work

Simulations in different science domains tend to become more and more complex and span over multiple disciplines and scales. These simulations usually have a large number of parameters to configure, and researchers spend a long time tuning the model parameters manually, which is difficult and time-consuming. To tackle these issues, it is necessary to have an automated tool that can be easily executed on local machines or likewise on super-computers. We present the L2L framework as a flexible tool to optimize and explore ranges of parameter spaces. Because the tool does not require a particular type of simulation, i.e., it is agnostic to the model in the inner-loop, it enables the optimization of any type of parameter resulting from the model, as long as fitness can be calculated and sent to the outer loop.

In Section 3, we described several neuroscientific use cases at different scales. The optimizations range from finding the correct set of parameter configurations to determining network dynamics to solving optimization problems up to exploring values for specific growth rules. In all cases, the optimization methods in the outer loop treated the inner loop simulations as black box problems and similarly, the optimization technique was unknown to the inner loop.

In terms of implementation, every optimizee follows the same structure by providing three functions: 1. creating the individual, i.e., the parameters to optimized, 2. starting and managing the optimizee run and providing fitness to asses the simulation performance, and 3. optionally constraining the parameter exploration range. The framework offers a plethora of built-in optimization techniques. Most of them are population based optimizers, which require several individuals and fitness or a fitness vector. Both the fitness and the population approach are incorporated into the optimization. For example, with genetic algorithms and the EnKF, the fitness is used to rank the individuals. A large population enables a wider range to explore parameters and find possible good initializations, which leads to a faster convergence. In order to not get stuck in local optima, most of the optimizers offer techniques to perturb the individuals and additionally enlarge the parameter space (which of course can be bounded if needed).

Clearly, executing a high number of individuals leads to an increase in computational requirements. By utilizing MPI in combination with the JUBE back-end, it is easy to deploy simulation and optimization on high performance computers in an automated fashion. From the users' perspective, only a few parameters have to be configured in a run script. The optimizees for the inner loop are created and the simulations are executed in parallel. One of the practical reasons for the population based optimizers is that the simulations are very easily parallelizable: each simulation can be conducted independently. Only the parameters have to be collected in a single step and fed into the optimizer. Afterward, the optimized parameters are distributed for the next generation and the new simulations can be started.

The TVB use case is an example of demonstrating a parallelized simulation in the optimizee. We show that we successfully reconfigured the gradient ascent optimizer to a version that can process a vector of fitnesses. We used this optimizer to find the best parameter setting for a TVB model such that the match between simulated functional and structural connectivity is optimal. Results from performance testing for the RateML (van der Vlag et al., 2022) models show that for a double state model such as the G2DO, on a GPU with 40 GB of memory, up to ≈62, 464 (61 times more parameters), can be simulated in a single generation, taking approximately the same amount of wall time due to the architecture of the GPU. This would reduce the time it takes for each generation and increases the range and resolution of the to be optimized processes even further; opening up possibilities for experiments requiring greater computational power. Moreover, this particular optimizer is not limited to TVB simulations only. Any process which uses a parallel architecture, e.g., GPU, CPU or FPGA, for which the output is a vector of fitnesses, can be adapted as an optimizee for the MGA optimizer. The utilization of the subprocess library and information transfers *via* in- and output text files, makes usage of this optimizer generic for any process. The MGA is just one example of an optimizer adapted to process multiple fitnesses, in theory, any of the optimizers can be adjusted to handle multi fitness optimizees.

### 4.1. Choice of Fitness Function and Optimizer

One important point to mention is the challenge of creating the fitness function. Every fitness function is a problem specific and finding a suitable function is often a complex task. In some cases, the fitness is given by the design of the problem (c.f. Section 3.1, in this case supervised learning). To illustrate the point, the task in Section 3.3 can be extended so that the ants are punished whenever they collide. However, just adding a simple cost value for the collision makes the training and optimization much harder, the ants exhibit erratic behaviors, such as spinning around or stopping moving after a few steps. Potentially, this behavior might resolve with enough generations, but it is more likely that the fitness function would need to be adapted. Even for the simple example shown here, the fitness function had to be carefully balanced in terms of the punishment and reward cost, which lead to several trials and manual adjustments. Thus, the exploratory and exploitative behavior is influenced by the fitness function. With a strict fitness function, i.e., every action in the simulation generates a reward or a punishment, it may be possible to exploit local optima; however, it may restrict the exploration of different, better optima. Conversely, making the fitness function too lax may lead to an overly exploratory behavior that does not exhibit any exploitation.

The choice of the optimizer is based on experience, the familiarity with the task and often includes a trial and error approach. Furthermore, the choice may be dependent on the task itself. For instance, in a supervised learning scheme, the “observable” parameter of the EnKF can be modified to support labels and enable this optimizer for supervised training. However, other optimization techniques may not be suitable as they cannot incorporate the concept of labels into their optimization process without extensive changes. It is not easy to recommend general optimization solutions for a variety of problems, and it is out of the scope of this work, we instead refer here to further literature (Okwu and Tartibu, 2020; Malik et al., 2021; Oliva et al., 2021). However, we would like to discuss some pointers which may be helpful in choosing an optimization technique when using L2L. Gradient descent and Kalman filtering can provide a directed and fast search within the parameter space. If it is known that the optimization problem space is smooth and ideally convex, the *gradient descent algorithm* is known for providing an efficient solution. The EnKF can also provide a fast convergence for non-convex problems with several optima and is especially suited for problems where calculation of the gradient is not possible or requires complex approximations. This can be particularly useful for problems where fast optimization with adequate results is more important than thorough explorations of vast parameter spaces to identify the optimal parameter configuration. Both the EnKF and gradient descent are suitable for optimization in high dimensional parameter spaces, such as the weight optimization of neural networks.

In contrast, if the solution space is not known and exploration is the focus, *genetic algorithms*—from the family of optimizers inspired by nature—may be the correct choice. By creating new individuals using mutation and cross-over, genetic algorithms can cover a vast space and still be very performant. For example, we also used genetic algorithms to optimize the network in use case 1 and obtained reasonable optimization results but did not reach as high a performance as with the ensemble Kalman filter (data not shown). The dimensionality of the parameter space in combination with the optimization algorithm chosen plays a key role in the outcome of the optimization. From our experience with the use cases presented here, we have seen that genetic algorithms work well with parameter spaces in the range of tens to thousands of dimensions.

Learning to learn provides several additional optimizers beyond those introduced in the use cases, which also have advantages in certain applications. The *evolution strategies* optimizer creates new individuals by perturbing, i.e., adding Gaussian noise, to the fittest individuals to create new ones and falls into the same category as the GA but uses stochastic gradient descent as an optimization technique. For example, the authors of Salimans et al. (2017) optimize large networks which are then able to play Atari games. Similarly, the *natural evolution strategies* (NES) optimizer samples from a multivariate Gaussian distribution to obtain new individuals. Wierstra et al. (2014) employ NES on several benchmark tasks with different parameter dimensions. They conclude that NES is applicable on low dimensional and high-dimensional and multi-modal problems.

The performance of *simulated annealing* depends heavily on the annealing schedule selected. L2L provides a variety of schedules to choose from the exploration progress and they define the ratio between exploration and exploitation of the algorithm. Simulated annealing can be an excellent tool to perform initial explorations of large parameter spaces and progressively move from exploration to exploitation as experience with the simulated model increases. The L2L version includes a cooling factor that allows the user to explore the balance between exploration and exploitation.

*Cross entropy* is highly directed and fast to converge. It is well suited for dealing with noisy optimization problems and large parameter spaces. In contrast, L2L also provides the *grid-search*, a technique that just iterates over the given parameter range in a brute force manner. This technique can be used for rather small parameter ranges if nothing is known about the problem space.

### 4.2. Outlook

Specifically regarding our presented use cases, future work will include multi-objective optimization to decouple the objectives from a specific fitness function and optimize the fitness functions in interchangeable steps. The L2L framework already supports multi-objective optimization since it can handle several fitness values. Alternatively, the optimizee can be written in such a way that it exchanges the fitness function in certain generations and still returns one fitness value.

A visualization of the trajectories through generations may give further insights for a follow-up analysis of the parameters. We aim to implement a visualization tool that can plot the evolution of the parameters using simple diagrams such as histograms, correlations, and similar statistics. A desirable feature would be to interact with the plot while the simulation is ongoing, as demonstrated by Tensorboard^7^. A challenge here is to interact with the results whenever the run is conducted on an HPC, as many super computing centers no longer allow X-forwarding—a network protocol to control and display a remote software from a local computer. Instead, other mechanisms for interactive computing need to be considered such as virtual network computing^8^.

In preliminary work, we were already able to run the L2L simulations on an HPC while instructing the run from a local machine. By utilizing UNICORE (Streit et al., 2005), a tool for distributed computing, we could successfully send an optimizee to a specified HPC, initialize the L2L framework, run the optimizations, and collect the results. For this approach to work, we have to ensure that the L2L framework is correctly deployed on the remote side. Seamless integration of all tools in the process chain is required. This approach also leads toward a vision of L2L as a service, where users can submit optimization workloads using a simple API. Despite the advantages of this approach, new aspects should be considered to protect user data and any sensitive data that can be used or produced during simulations. In order to deploy this service, full integration with the EBRAINS^9^. infrastructure is our target for the near future, as this will enable L2L to support the neuroscience community while being part of a well-established research platform.

Another necessary element, which is currently only available in a preliminary form, is check-pointing the run, i.e., the possibility to continue the inner and outer loop processes to a later time. This would allow us to execute jobs in a very long period without any HPC time restriction. At the moment, the run-script (see Section 2.3) has to be changed with a few more routines to load the trajectories from an earlier run and to continue it. In an upcoming release, this component will be integrated into the L2L framework.

Finally, we would like to extend the set of optimization techniques with optimizers that have more capabilities. This would be for example a neural network, along the lines of the approach proposed by Andrychowicz et al. (2016). For instance, the network could learn the distribution of the parameter space and predict the next set of parameters. One other interesting direction is to include Bayesian Optimization *via* Bayesian hierarchical modeling. In this case, the parameters are not optimized directly as depicted in this work, instead, uncertainty measures and prediction uncertainty are inferred (Finn et al., 2018; Gordon et al., 2018; Yoon et al., 2018).

In conclusion, with this work, we have presented L2L as a software framework for the hyper-parameter optimization of computing workloads, especially focusing on neuroscience use cases. The flexibility of this framework is designed to support the broad and interdisciplinary nature of brain research and provides easier access to HPC for ML-based optimization tasks.

## Data Availability Statement

Publicly available datasets were analyzed in this study. This data can be found here: The Modified National Institute of Standards and Technology (MNIST) database, http://yann.lecun.com/exdb/mnist/.

## Author Contributions

AS, AY, WK, and SD-P worked on the design of the framework. AY, AS, SD-P, and WK worked on the implementation. AY, TH, CJ-R, WK, AP, MV, and SD-P implemented the use cases and produced the results reported in the manuscript. All authors conceived of the project, designed the set of use cases, reviewed, contributed, and approved the final version of the manuscript.

## Funding

The research leading to these results has received funding from the European Union's Horizon 2020 Framework Programme for Research and Innovation under the Specific Grant Agreements no. 785907 (Human Brain Project SGA2) and 945539 (Human Brain Project SGA3). This research has also been partially funded by the Helmholtz Association through the Helmholtz Portfolio Theme Supercomputing and Modeling for the Human Brain. Open Access publication funded by the Deutsche Forschungsgemeinschaft (DFG, German Research Foundation)-491111487.

## Conflict of Interest

The authors declare that the research was conducted in the absence of any commercial or financial relationships that could be construed as a potential conflict of interest.

## Publisher's Note

All claims expressed in this article are solely those of the authors and do not necessarily represent those of their affiliated organizations, or those of the publisher, the editors and the reviewers. Any product that may be evaluated in this article, or claim that may be made by its manufacturer, is not guaranteed or endorsed by the publisher.

## Footnotes

^1^ https://github.com/Meta-optimization/L2L

^2^ https://slurm.schedmd.com/

^3^ https://github.com/SmokinCaterpillar/pypet

^4^Note that this can introduce different issues with numerical precision if said ranges span too many orders of magnitude.

^5^ https://docs.python.org/3/library/subprocess.html

^6^ https://fz-juelich.de/ias/jsc/EN/Expertise/Supercomputers/JUSUF/JUSUF_node.html

^7^ https://www.tensorflow.org/tensorboard/

^8^ https://trac.version.fz-juelich.de/vis/wiki/vnc3d

^9^ https://ebrains.eu/

## Supplemental Data

All code used to produce the results in this paper as well as the L2L framework can be accessed in this repository: https://github.com/Meta-optimization/L2L/tree/frontiers_submission.

Installation instructions for the framework can be found in the README file of the repository.

An additional use case using structural plasticity in NEST can be found in the Supplementary Material.
