# Supplementary material for: Exploring Parameter and Hyper-Parameter Spaces of Neuroscience Models on High Performance Computers With Learning to Learn
Source: Front Comput Neurosci. 2022 May 27;16:885207. doi: 10.3389/fncom.2022.885207 (PMC9199579; doi:10.3389/fncom.2022.885207)
Supplement: Supplementary file 1 [file Data_Sheet_1.pdf]

frontiers

Supplementary Material

1 SUPPLEMENTARY USE CASE: OPTIMIZING STRUCTURAL PLASTICITY IN NEST

Structural plasticity is the ability of neurons to change their morphology in order to adapt to stimuli and reach specific activity regimes. It depends on the ability of each neuron to create and remove synaptic connections with other neurons in the network. It is a fundamental component of brain development, learning, healing after lesions and adaptation. A network model with structural plasticity will change its connectivity through time, depending on a set of rules which are local to each neuron and regulate the generation or deletion of abstracted boutons and spines.

Note that the structural plasticity algorithm searches the connectivity space of the whole network in a multi objective optimization process, where all neurons aim at reaching a specific firing rate by creating an appropriate set of connections. Structural plasticity works as a controller of the structure of the network aiming at reducing the error between the target activity set point of each neuron and its current activity. The definition of these control guidelines is consolidated in the growth rules, thus their shape is very important to achieve the desired target. The growth curve determines how fast and how strongly the structural changes will take place. An inappropriately growth curve can lead to high oscillations in the activity of the network, taking it to an unstable regime. Delayed structural changes with respect to the error signal can also result in a system that never converges to stable activity.

The goal of this task is to use L2L to optimize the growth rules that guide the structural plasticity algorithm implemented in NEST, which is based on a homeostatic principle: neurons that have an average activity below their set point will create new connections, and over-active neurons will delete them, according to a user-defined growth curve. Frequently used growth curves are of linear and Gaussian shape. Here, we make use of a Gaussian growth curve, which are defined by three parameters: the target firing rate ϵ, the minimum start firing rate η, and the growth rate ν. The variable to optimize for each growth curve in this case is the growth rate ν.

1.1 Description of the simulation tools

In this use case we use NEST (see Section 3.1.1 of the main manuscript) and its implementation of structural plasticity. This implementation is based on the model by Butz and van Ooyen (2013) in which neurons are able to grow and rescind synaptic elements representing boutons and spines. Compatible synaptic elements can be chosen to create a new synapse when they become mature. If a synaptic element in an existing synapse is rescinded, then the synapse is deleted but the counterpart element can be rewired in a new synapse. The growth of the synaptic elements is guided by homeostatic rules which can be defined by the user and can differ between populations or even neurons. A comprehensive description of the structural implementation algorithm can be found in Diaz-Pier et al. (2016).

1.2 Optimizee: Spiking neural networks with structural plasticity

In this use case we have two structural plasticity optimizees. The first optimizee consists of a simple two population model: the inhibitory population contains 20% of the neurons in the network while the excitatory population contains the other 80% (see Figure S1A. The second optimizee is an implementation of the cortical microcircuit model proposed by Potjans and Diesmann (2012). This model has eight populations,

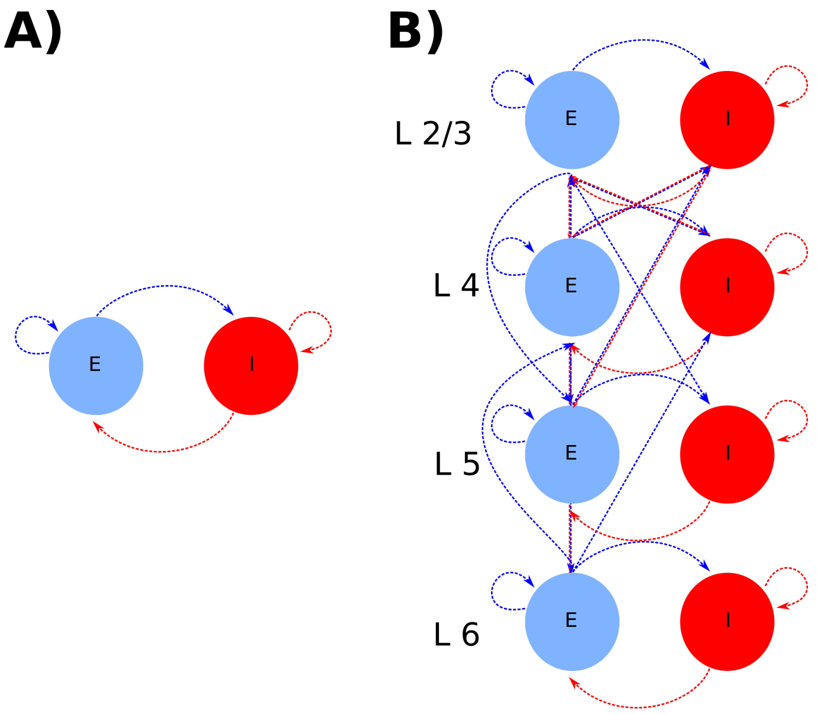

Figure S1. Spiking neural network models with structural plasticity. **A)** Simple two population model, and **B)** the cortical microcircuit model.

four excitatory and four inhibitory, in a layered fashion representing the cortical layers $2/3$, 4, 5, and 6 (see Figure S1B.

In the case of the two population model, the network is simulated for a total of 200 cycles of 2 s of biological time each. The task is to find a value for the growth rate ν which enables the network to reach target activity ranges for both populations within that time: between 5 and 15 spks/s for the inhibitory population and between 1 and 4 spks/s for the excitatory population.

For the microcircuit model optimizee the network is simulated for a total of 500 cycles of 1 s of biological time each. The task is to find growth rates for excitatory and inhibitory neurons (ν_e and ν_i) which allow all populations to grow connections bringing them to their desired activity regimes within this time. Each population has a different target firing rate according to the experimental recordings used to constrain the original model, see Potjans and Diesmann (2012).

Each job run provides a parallel set of initial states. Inner loop simulations are 200 s long because the network starts without any connections besides a Poissonian background noise and the timescale for structural plasticity is slow (order of seconds in our implementation) with respect to other neuronal timescales such as the membrane time constant. At the end of each simulation, the average firing rate of all neurons is collected in order to evaluate the fitness, see below.

All experiments were performed using NEST 2.18 Jordan et al. (2019) on the JURECA supercomputer of the Jülich Supercomputing Centre in Jülich, Germany.

1.3 Fitness metric

In order to assess the performance of each instance of the network with specific parameters, a multiobjective fitness rule was derived. Several compound fitness measures were tested for the multi-objective optimization, resulting in a weighted measure which gives more importance to the fitness of the excitatory than the inhibitory neurons because of their larger presence in the network.

The fitness metric used for the simple model is defined as:

$$f = \frac{1}{0.8 * (\lambda_{\mathrm{e}} - \epsilon_{\mathrm{e}}) + 0.2 * (\lambda_{\mathrm{i}} - \epsilon_{\mathrm{i}})}, \tag{S1}$$

where $\lambda_{\mathrm{e/i}}$ is the average firing rate of the excitatory (inhibitory) population at the end of the simulation and $\epsilon_{\mathrm{e/i}}$ is the corresponding target firing rate. The choice of growth rate ν influences the number of connections created and the general activity of the network, leading to different simulation times. In order to constrain the simulations, the maximum time allowed was set to $20\,\mathrm{min}$ and simulations which did not finish (i.e. successfully simulate $2\ \mathrm{s}$ of biological time) within that period were penalized with the worst fitness (a value of 0).

In the case of the microcircuit optimizee, a compound rule similar to the one in the simple model was used:

$$f = \frac{1}{\sum_l (\lambda^l_{\mathrm{e}} - \epsilon^l_{\mathrm{e}}) + (\lambda^l_{\mathrm{i}} - \epsilon^l_{\mathrm{i}})}, \tag{S2}$$

where $\lambda^l_{\mathrm{e/i}}$ and $\epsilon^l_{\mathrm{e/i}}$ are the average (of all neurons in a population at the end of the simulation) and target firing rates, respectively in the excitatory (inhibitory) population of layer l, and $l \in \{2/3, 4, 5, 6\}$. As with the two-population model, a time limit was placed on the simulations. Any simulation that did not successfully simulate X biological seconds in $60\,\mathrm{min}$ of simulation was evaluated to have a fitness of 0

1.4 Optimizers

For the simple two populations optimizee, the outer loop optimization algorithms were evolutionary strategies, gradient descent and simulated annealing. For the microcircuit model optimizee, the outer loop algorithm chosen was gradient descent.

1.5 Analysis

Figure S2 shows the results of executing the simple optimizee in different scenarios. In panel A) we can see that the simulated annealing algorithm performs a homogeneously distributed search on the space. This has the advantage that local minima are easier to avoid. The algorithm still focuses its search efforts in the lower right corner of the parameter space but uses some of the exploratory resources on areas of lower interest. In the case of structural plasticity, simulated annealing is a good candidate to perform initial explorations of vast parameter spaces which provide a good idea of the shape of the space and where areas of interest are located. The cross entropy algorithm is very effective in finding the area of interest very fast as shown in panel B). This makes best use of computational resources but might miss other interesting areas of the parameter space to be explored. Finally, the gradient descent algorithm, as shown in panel C) presents a middle ground between simulated annealing and cross entropy in terms of exploration and focus. In all three algorithms we can see that there are parameter combinations outside of the lower right corner which have high fitness. The random nature of the structural plasticity algorithm and the task definition can also cause variability in the performance of the model and thus make the exploration more challenging.

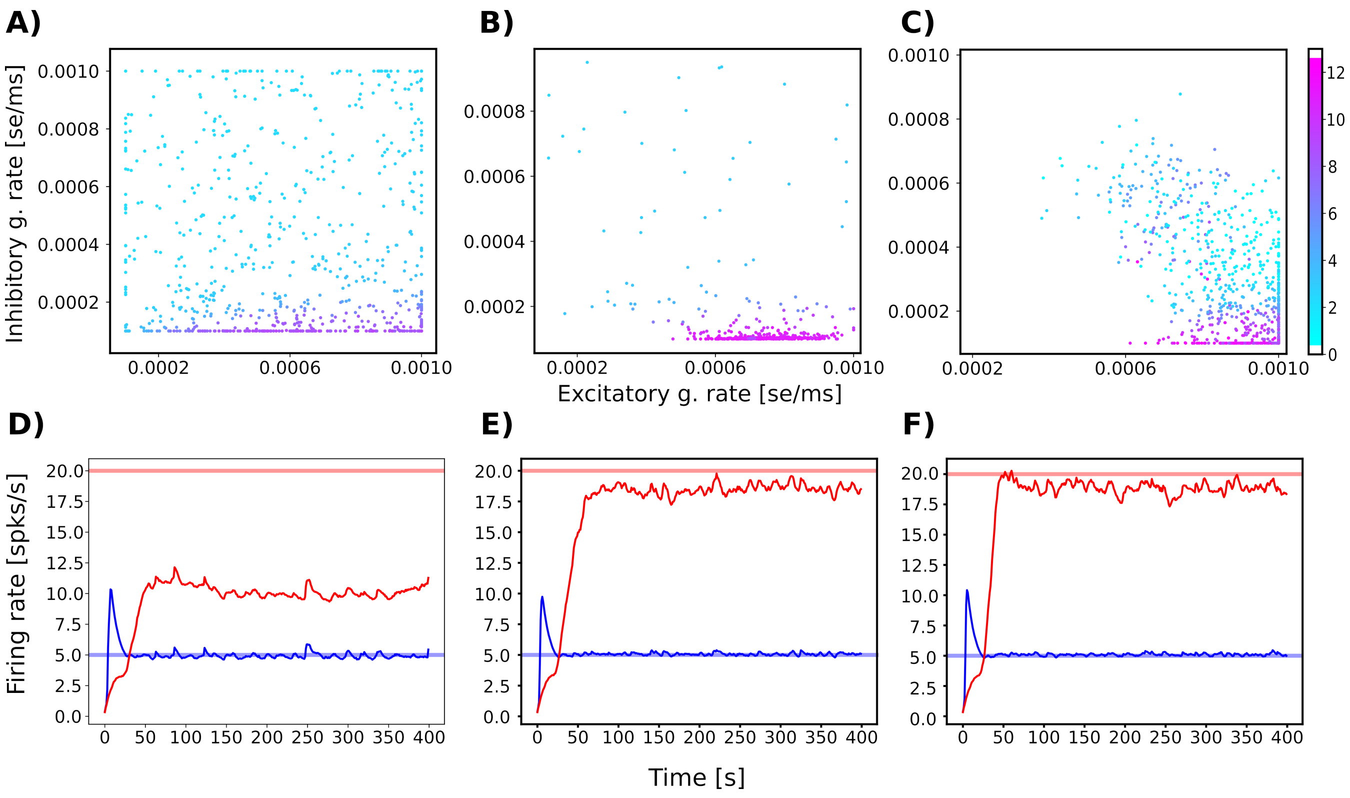

Figure S2. Parameter exploration for the two population optimizee using **A)** gradient descent, **B)** simulated annealing and **C)** cross entropy as outer later optimizers. Each dot in the plot represents a position in the parameter space described by the inhibitory growth rate on the y-axis and the excitatory growth rate in the x-axis. The color of the dot indicates the fitness of that parameter combination. The performance of the best individual using gradient descent at generations 1, 5 and 20 can be seen in panels **C)**, **D)**, and **E)** correspondingly. Blue and red curves represent the average firing rate of the neurons in the excitatory and inhibitory populations accordingly. The light blue and red horizontal lines represent the target firing rates for the excitatory and inhibitory populations, respectively.

S2D), E) and F) show the best individual for generations 1, 5, and 15 using gradient descent. The network converges to better solutions more quickly as the gradient descent algorithm finds more effective structural plasticity growth parameters.

In Figure S3 A) we can see the parameter exploration for the microcircuit model. Panel B) shows the best individual for the parameter exploration for the microcircuit model at generation 25. The best individual in generation 25 has an inhibitory growth rate of $0.0029\ \mathrm{se/ms}\ (\mathrm{synaptic\ elements/ms})$ and an excitatory growth rate of $0.0030\ \mathrm{se/ms}$. The results show that this model benefits from having growth rate values between excitatory and inhibitory neurons which are more similar to each other. Gradient descent is an algorithm which is able to explore well the parameter space of the growth rules but also enables fast convergence. The parameter space in this case seems to be smooth because the fitness increases gradually towards the point of best performance without many irregularities. Because of this, gradient descent proves to be a good choice.

The simple metrics used in these use cases worked well but more complicated models might need to take into account other features of the network activity and normalise with respect to the target rate of each population.

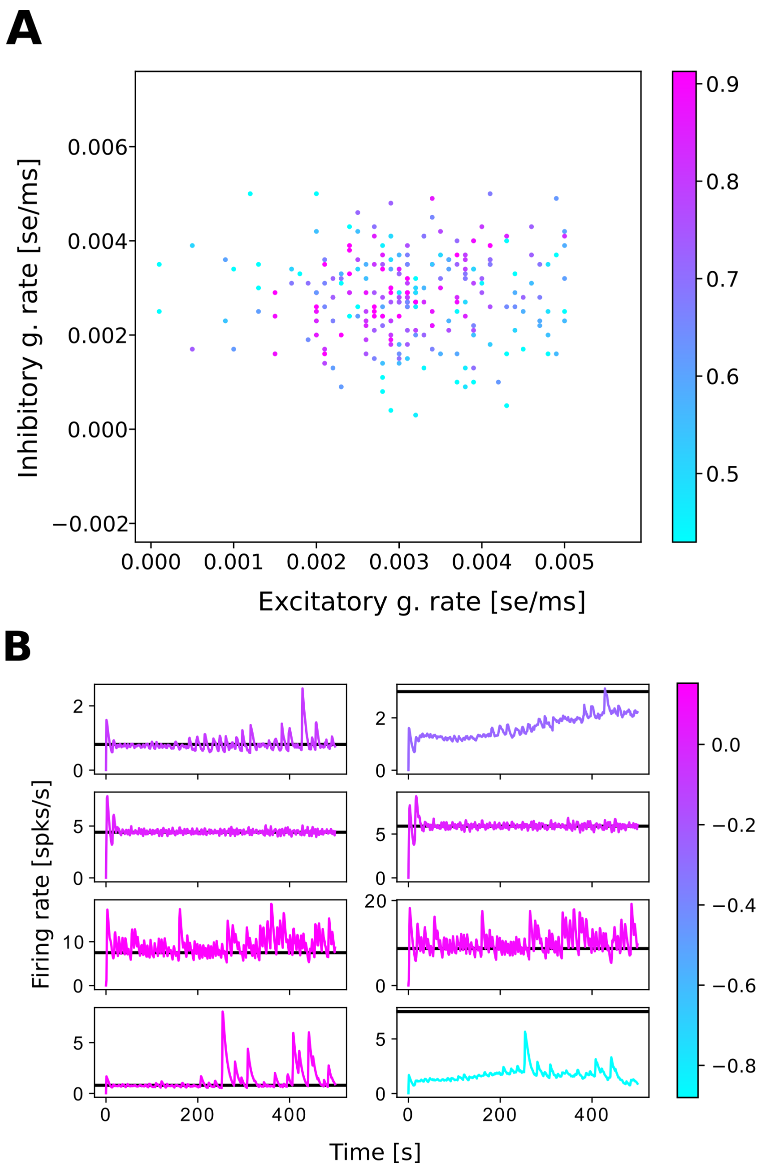

Figure S3. A) Parameter exploration for the cortical microcircuit task. B) Best individual during the parameter exploration for the cortical microcircuit task after 25 generations. The position of each subpanel corresponds to the position of each population in the model diagram in Figure S1 B. The color of the line reflects the normalized difference between the average firing rate in each population at the end of the simulation and the target firing rate. The black horizontal lines mark the target firing rate of each population.

The best parameter configuration depends on the flexibility we give to the model (e.g. which connections are plastic and which network / cell parameters can be changed) as well as the functional and structural constraints we provide to the outer loop algorithm (e.g. limits on total numbers of synapses per neuron or minimum ration between excitation and inhibition). This is a more complex model which could benefit from exploring some variations. For example, we could use the power spectrum of the activity in the model to define the fitness. Different plasticity rules per layer could also be defined to enhance the flexibility of the model. The L2L framework provides a platform to do large parameter explorations with adaptive algorithms, reducing time compared to brute force approaches. Visual exploration of plasticity parameter spaces, as discussed in Nowke et al. (2018), is very useful to find general areas of interest and understand the

relationships between variables. However, once this understanding is acquired, it can be further translated into good fitness functions which allow for a systematic exploration of the space with high throughput on HPC. Structural plasticity simulations require long simulation times because the structural changes take place at a slow rate. This is particularly critical as the networks become more complex or other plasticity rules are also in place. Finding optimal ways to explore the parameter space is crucial to reduce wasting computational resources on areas of no interest or biological relevance.

Structural plasticity is not a frequently simulated phenomenon in computational neuroscience. There are few simulators and emulators capable of creating and deleting synapses while the dynamics of the network are being computed. Other reports in literature only refer to visual exploration of these hyperparameters as reported in Nowke et al. (2018), where the performance of the selected plasticity rules varies with the trajectory selected by the user.

2 APPENDIX

2.1 Summary of use cases

Use Case	Optimizer	Parameters	Hyper-parameter
1: Reservoir	ensemble Kalman filter	connection weights	
2:Arbor	genetic algortihm	sub-section of the morphology<br>ion-channel id<br>parameter name<br>value to set the parameter to	
3: Ants	genetic algorithm	connection weights<br>delays	
4: TVB	gradient ascent	coupling strength<br>connection speed	
5: Gym	genetic algorithm	connection weights and delays	
6: Plasticity	cross-entropy<br>simulated annealing<br>gradient ascent		synaptic growth rates

Table S1. The table summarizes the optimizer and the optimized (hyper-) parameters per use case presented in the Results Section of the main manuscript. However, when optimizing the L2L-framework makes no conceptual distinction between hyper- and parameter.

2.2 Getting started with L2L

This section describes how to run a general L2L simulation run from the user perspective. To develop an optimizer we refer to the documentary in the GitHub repository. The workflow to obtain the results presented in Section 3 of the main manuscript is documented at `https://github.com/Meta-optimization/L2L/tree/frontiers_submission`.

2.2.1 Installing the package

- Clone the repository from `https://github.com/Meta-optimization/L2L/`
- To install the package execute `pip install . -e` from within the directory. This install the package in a editable mode. This is useful when implementing an optimizee or optimizer. Changes in the base code will be directly reflected in the installed package without reinstalling it.
- At a later version we will provide a `PyPI` package to automatically install from the Python package manager without cloning the repository.

2.2.2 Setting up an optimizee

The optimizee defines the individuals to be optimized. The model has to be defined here as well. L2L allows to execute sub-processes if the model needs to access libraries outside the framework. It is important to note that the optimizee is stateless, after a the parameters and fitness are sent to the optimizer the optimizee object state is not accessible anymore. In the next generation the optimizee will be re-created. The optimizees are located at `l2l/optimizess/`.

- Three functions have to be implemented in the optimizee.
  1. `create_individual`: Defines the (hyper-) parameters to be optimized. A parameter would be e.g. weights, a hyper-parameter e.g. the learning rate. In L2L both parameters can be optimized.
  2. `simulate`: This function executes the model. After the simulation the fitness, i.e. the performance of the run, has to calculated and returned as a Python tuple object.
  3. `bounding_func`: An optional function to define boundary conditions. E.g. delays should be strictly positive or parameters should not exceed certain ranges.
- The optimizee can accept parameters as well, e.g. parameters for the model (which are not optimized). They have to be defined as a Python `namedtuple` object and will be passed from within the run script.

2.2.3 Run scripts

Run scripts initiate the whole L2L run. Exemplary scripts can be located and new ones should be saved in the `bin` folder. A template script was thoroughly described in Section 2. Here, we will summarize the steps.

- The optimizee, optimizer and the experiment class have to be imported.
- The experiment class steers the run and initializes the trajectory, i.e. an object which communicates between the optimizer and optimizee and contains the history of the parameters.
- Parameters to the optimizee can be set here as well.
- In L2L common parameters such as the population size need to be specified for the optimizer. Additionally, hyper-parameters specifically for the optimization have to be set, too. For instance, the mutation rate for the genetic algorithm.
- The path where the results will be saved need to be given as well. The parameters contained in the trajectory will be saved in <path_to_results>/trajectories, fitness values can be found in <path_to_results>/results. In the <path_to_results>/ folder additional files are stored which are important for JUBE (see Section 2 of the main manuscript).
- Finally, if the run is conducted on an HPC parameters for the queuing system can be specified as well.

2.2.4 Troubleshooting

Here is a list of things that should be checked when troubleshooting optimizees

- The `create_individual` and `bounding_func` need to be passed to the optimizer class constructor in the L2L run script.
- Make sure to specify the *exec* parameter with the right command to execute L2L optimizees: either mpriun or srun depending on the MPI installation, or just python in case of local sequential executions.
- The results of executing each optimizee are stored in specific folders for each individual and generation. Exploration of logs from specific executions and optimizees can be achieved by looking into the results

folder defined in the L2L execution scripts. Standard output and errors can be found in the results folder at <path_to_results>/simulation/work/jobsystem_bench* for every generation and individual. However, errors can be propagated to the main L2L script standard output if a variable `debug=True` is provided as a parameter for the function `prepare_experiment` in the run script.

- Normalization can be useful if the optimized parameters are in different ranges. This has to be done in the optimizee before sending the parameters into the optimization process. Eventually, a re-normalization is needed after receiving the optimized parameters in the optimizee.
- It is always recommended to do a test execution of the optimization process at a low scale before launching long optimizations which use large amounts of computing resources.
- When optimization runs seem not to provide the desired optimization outcome, please check that the parameters of the optimizer are correctly set. These parameters may need to be adjusted based on experience with each optimizee.
- It is possible to launch optimizees which are encapsulated in containers and virtual machines. Please make sure that the user who launches the optimization has read and write permissions on all required components required for the optimizee execution.

REFERENCES

Butz, M. and van Ooyen, A. (2013). A simple rule for dendritic spine and axonal bouton formation can account for cortical reorganization after focal retinal lesions. *PLoS Comput Biol* 9, e1003259

Diaz-Pier, S., Naveau, M., Butz-Ostendorf, M., and Morrison, A. (2016). Automatic generation of connectivity for large-scale neuronal network models through structural plasticity. *Frontiers in Neuroanatomy* 10. doi:10.3389/fnana.2016.00057

[Dataset] Jordan, J., Mørk, H., Vennemo, S. B., Terhorst, D., Peyser, A., Ippen, T., et al. (2019). Nest 2.18.0. doi:10.5281/zenodo.2605422

Nowke, C., Diaz-Pier, S., Weyers, B., Hentschel, B., Morrison, A., Kuhlen, T. W., et al. (2018). Toward rigorous parameterization of underconstrained neural network models through interactive visualization and steering of connectivity generation. *Frontiers in neuroinformatics* 12, 32

Potjans, T. C. and Diesmann, M. (2012). The Cell-Type Specific Cortical Microcircuit: Relating structure and activity in a full-scale spiking network model. *Cerebral Cortex* 24, 785–806. doi:10.1093/cercor/bhs358
